# The Construction of Bone Metastasis-Specific Prognostic Model and Co-expressed Network of Alternative Splicing in Breast Cancer

**DOI:** 10.3389/fcell.2020.00790

**Published:** 2020-08-25

**Authors:** Runzhi Huang, Juanru Guo, Penghui Yan, Suna Zhai, Peng Hu, Xiaolong Zhu, Jiayao Zhang, Yannan Qiao, Yu Zhang, Hui Liu, Ling Huang, Jie Zhang, Daoke Yang, Zongqiang Huang

**Affiliations:** ^1^Department of Orthopedics, The First Affiliated Hospital of Zhengzhou University, Zhengzhou, China; ^2^Division of Spine, Department of Orthopedics, Tongji Hospital Affiliated to Tongji University School of Medicine, Shanghai, China; ^3^Tongji University School of Medicine, Shanghai, China; ^4^Tongji University School of Mathematical Sciences, Tongji University, Shanghai, China; ^5^Department of Radiotherapy, The First Affiliated Hospital of Zhengzhou University, Zhengzhou, China

**Keywords:** breast cancer, alternative splicing event, splicing factor, prognostic model, pathway, bone metastasis

## Abstract

**Background:**

Breast cancer (BRCA) ranks among the top most common female malignancies and was regarded as incurable when combined with bone and distant metastasis. Alternative splicing events (ASEs) together with splicing factors (SFs) were considered responsible for the development and progression of tumors.

**Methods:**

Datasets including RNA sequencing and ASEs of BRCA samples were achieved from TCGA and TCGASpliceSeq databases. Then, a survival model was built including 15 overall-survival-associated splicing events (OS-SEs) by Cox regression and Lasso regression. The co-expressed SFs of each bone-and-distant-metastasis-related OS-SE were discovered by Pearson correlation analysis. Additionally, Gene Set Variation Analysis (GSVA) was performed to identify the downstream mechanisms of the key OS-SEs. Finally, the results were validated in different online platforms.

**Results:**

A reliable survival model was established (the area under ROC = 0.856), and CIRBP was found co-expressed with FAM110B (*R* = 0.320, *P* < 0.001) associated with the fatty acid metabolism pathway.

**Conclusion:**

Aberrant SF, CIRBP, regulated a specific ASE, exon skip (ES) of FAM110B, during which the fatty acid metabolism pathway played an essential part in tumorigenesis and prognosis of BRCA.

## Introduction

Breast cancer (BRCA) ranks among the top most common female malignancies in the world (Harbeck et al., 2019). Surgery of the primary tumor remains a cornerstone of curative breast cancer treatment (Mclaughlin, 2013). Systemic therapies for primary breast cancer are quite effective, and adjuvant chemotherapy and adjuvant endocrine therapy are able to decrease the mortality of breast cancer by one third (Early Breast Cancer Trialists’ Collaborative Group, Davies et al. 2011, 2012). Unfortunately, advanced BRCA is a virtually incurable disease, while metastases are the cause of death in almost all patients, the median overall survival of it is 2–3 years, and the common sites of spread are the bone (most frequent cite), the lungs, and the liver (Cardoso et al., 2018). In order to improve the prognosis of BRCA more efficiently, it is an urgent need to explore the pathogenic mechanism in metastasis and prognosis.

Alternative splicing (AS) took place in human genes quite commonly (Wang et al., 2008) and was critically associated with the carcinogenic process (Zhao et al., 2020) during which splicing factors (SFs) play key roles. Dysregulating the network built by SFs and alternative splicing events (ASEs), the aberrant AS for some genes and somatic mutations of SFs have been reported that they would cause epithelial–mesenchymal transition and might modulate malignant transformation of cells (Sveen et al., 2016; Kouyama et al., 2019; Wu et al., 2019; Xing et al., 2019). Identification of the aberrant regulation network of SFs and ASEs could not only help predict prognostic and metastatic molecular biomarkers but also find out the potential therapeutic targets (Lee and Abdel-Wahab, 2016; Wang et al., 2019). Most previous studies of BRCA concentrated on alteration at the transcriptome level, but no researchers have studied the analysis of the posttranscriptional process so far.

In this study, based on an all-round analysis of the BRCA dataset, we identified overall-survival-related alternative splicing events (OS-SEs) using Pearson correlation analysis and established a prognostic model by Cox regression accordingly. We then detected the metastasis-related ASEs and their corresponding co-expressed, survival-related SFs and pathways of BRCA. Consequently, the prognostic model we constructed was of great significance for the prediction of BRCA prognosis; we also proposed a potential molecular mechanism and therapeutic target of BRCA.

## Materials and Methods

### Data Collection and Preprocessing

This study was approved by the Ethics Committee of The First Affiliated Hospital of Zhengzhou University. The RNA-seq data of the data used in this study could be downloaded in the Cancer Genome Atlas (TCGA) database^1^. Demographics, tumor information, and follow-up data of all patients were also retrieved from the database. Furthermore, the seven types of ASE data [alternate acceptor (AA); alternate donor (AD); alternate promoter (AP); alternate terminator (AT); exon skip (ES); mutually exclusive exons (ME); retained intron (RI)] along with the Present Spliced In (PSI) values were available in the TCGASpliceSeq database^2^ (Ryan et al., 2016). In order to have more reliable results, the data was cleaned under the following rules:

(1) Samples containing over 25 of the missing PSI values or without follow-up records were excluded, and the rest of the missing values were supplemented utilizing the K-nearest neighbor algorithm (*k* = 10).
(2) ASEs whose mean value of PSI less than 0.01 or standard deviations value of PSI less than 0.01 were ruled out.

### Independent Prognostic Model Construction

Upset plots were presented to display the relationship between the ASEs and genes directly. For further exploring the contribution of ASE on survival, the univariate Cox regression analysis was adopted using the clinical data to calculate the hazard ratio and its *p*-value of each filtered ASE. A volcano plot was adopted to show the prognosis-related as well as unrelated ASEs. The top 20 OS-SEs of each splicing pattern were screened according to *p*-value and were presented in Bubble plots. In addition, to prevent the overfitting of the prognostic model, the selected OS-SEs were used to perform Lasso regression, deleting highly correlated OS-SEs. Accordingly, the final significant OS-SEs were confirmed; *n* denotes the total number of the selection. The mentioned data of OS-SEs and patents’ living status were applied to the multivariate Cox regression model. Thus, the regression coefficient of the *jth* OS-SE was calculated, denoted as β_*OS*−*SEj*_,*j* = 1,2,…,*n*, and the PSI value of the *ith*sample and the *jth* OS-SE was represented as *PSI*_*OS*−*SEij*_,*j* = 1,2,…,*n*. Mathematically, the risk score (RS) of each sample can be described in the following equation:

$$R\,S_{i}=\sum_{j=1}^{n}\beta_{O\,S-S\,E\,j}\times P\,S\,I_{O\,S-S\,E\,i\,j}$$

In addition, the samples were then classified into the high-risk group as well as the low-risk group on the basis of the median value of the risk score, and the ROC curve was applied to demonstrate model accuracy. At last, to better compare the difference between the two groups, the survival curve of each group was estimated by Kaplan–Meier survival analysis and the risk curve, scatterplot, and expression heat map were demonstrated as well.

Consequently, both univariate and multivariate Cox regression analyses were applied to the processed dataset with age, gender, grade, stage, TNM stage, and risk score. Then, factors whose *p*-value of both univariate and multivariate Cox regression analyses were less than 0.05 were finally considered as independent prognostic factors.

### Discoveries of Relationship Between SF, Metastasis, Stage, and OS-SEs

The dataset with 390 SF factors was achieved from the SpliceAid2 database (Piva et al., 2009). The correlation coefficients and *p*-values between SFs and OS-SEs were calculated by Pearson correlation analysis to discover their relationship. The SFs and OS-SEs were selected if their absolute value of correlation was more than 0.3 and their *p* < 0.001. Metastasis information and TNM stage were pivotal paraments of breast cancer so the relationship with OS-SEs may be of great worth. The Kruskal–Wallis test and Mann–Whitney–Wilcoxon test were performed and drawn in Beeswarm plots.

Based on the analyses above, Cytoscape (3.7.1) (Shannon et al., 2003) was used to present the interaction of SF, metastasis, stage, and OS-SEs with high risk as red circles, low risk as purple circles, and the related SF as arrows and the lines indicating their relationships.

### Downstream Signaling Pathway Analysis

In order to better compare the results of enrichment analysis and conduct co-expression analysis, quantitative results were quite essential. In this case, Gene Set Variation Analysis (GSVA) (Hanzelmann et al., 2013) was adapted to find out the possible KEGG pathways in BRCA. Prognosis-related ones were selected by univariate Cox analysis. Based on the results above, KEGG pathways co-expressed with metastasis-related OS-SEs were discovered by Pearson analysis. In this way, the potential mechanism of the bone metastasis related to BRCA was identified.

### Multidimensional Validation

Genes in the core of the protein–protein interaction networks in ties with the KEGG pathway were detected by Pathway Card^3^. The results of the critical biomarkers were validated to reduce bias conclusions in different dimensions. The significant biomarkers above need external validation to reduce the false-positive rate. Then, a multidimensional validation was performed here. PROGgeneV2 (Goswami and Nakshatri, 2014), Gene Expression Profiling Interactive Analysis (GEPIA) (Tang et al., 2017), UALCAN (Chandrashekar et al., 2017), Linkedomics (Vasaikar et al., 2018), cBioportal (Cerami et al., 2012), and Kaplan–Meier plotter (Nagy et al., 2018) showed the relationship between key genes and patients’ survival. The Human Protein Atlas (Uhlen et al., 2015) and Genotype-Tissue Expression (GTEx) (Consortium, 2015) demonstrated the expression levels of genes and proteins at the tissue level. Oncomine (Rhodes et al., 2004) presented the results of a multi-study meta-analysis of key genes at the transcriptional level. The Cancer Cell Line Encyclopedia (CCLE) (Ghandi et al., 2019) illustrated the co-expression of the key genes at cellular levels, and String (Snel et al., 2000) displayed the network of SFs and OS-SEs and the key members of the potential pathway. More importantly, they validate in different omics levels. CCLE, cBioportal, and Linkedomics validate at the genomics level; cBioportal, CCLE, UALCAN, Linkedomics, GEPIA, PROGgeneV2, and SurvExpress validate at the clinical level. All datasets except String mentioned in this section perform at the transcriptomic level.

### Independent Dataset Validation and Spatial Transcriptome Validation

One independent dataset with a metastasis-free survival (MFS) endpoint was used for independent dataset validation (accession number: GSE11121)^4^ (Schmidt et al., 2008).

Additionally, the RNA binding proteins are involved in many biological processes such as RNA maturation, transport, localization, and translation. SFs are a special class of RBPs that can mediate alternative splicing. We have speculated that aberrant SF, CIRBP, regulated a specific ASE, exon skip (ES) of FAM110B, during which the fatty acid metabolism pathway might play an essential part in tumorigenesis, bone metastasis, and prognosis of BRCA. However, the regulation mechanism of SF-mediated AS needs to be demonstrated by molecular biological experiments (e.g., RNA Binding Protein Immunoprecipitation (RIP), Luciferase reporter gene assay). Therefore, spatial transcriptome combined with single-cell RNA sequence (scRNA-seq) data of BRCA was used to identify the cell subtype localization of the key genes in the speculative regulatory mechanism^5^. In terms of quality control (QC), genes with count greater than 1 and expressing in at least three single cells were considered for further analysis. Cells with either fewer than 100,000 transcripts or fewer than 1,500 genes were filtered out.

The Seurat method was applied for integrated data analysis (Butler et al., 2018). After the “sctransform” algorithm for normalization, the “vst” method was used to identify variable genes while the “markvariogram” method was performed to find spatial-specific genes. Variable genes were the input as initial features for principal component analysis (PCA) (Butler et al., 2018). Then, the principal components (PCs) with *P* < 0.05 were filtered by jackstraw analysis and were incorporated into further t-distributed Stochastic Neighbor Embedding (t-SNE), UMAP (Uniform Manifold Approximation and Projection), and Hematoxylin and Eosin (HE) staining slide to identify cell subclusters (resolution = 0.50) (Chung and Storey, 2015). Only the genes with | log2 fold change (FC)| greater than 0.5 and false discovery rate (FDR) value < 0.05 were selected as differentially expressed genes (DEGs) among cell subclusters. The (spatial) feature plots and violin plots were utilized to illustrate the distribution and expression of DEGs, respectively. Additionally, scMatch (Hou et al., 2019), singleR (Aran et al., 2019), and CellMarker (Zhang X. et al., 2019) databases were used as references for defining each cluster. Furthermore, 50 hallmark gene sets were retrieved from the Molecular Signatures Database (MSigDB) v7.1^6^ and Gene Set Variation Analysis (GSVA) was performed to absolutely quantify the signaling pathway activity in each single cell (Hanzelmann et al., 2013; Liberzon et al., 2015).

### Statistical Analysis

Statistical analyses were performed on R 3.6.1 software (R Foundation for Statistical Computing, Vienna, Austria)^7^ with packages of impute, glmnet, UpSetR, survminer, survivalROC, ggplot2, rms, forestplot, beeswarm, and preprocessCore. *P* < 0.05 were considered statistically significant except for the selection of SFs and OS-SEs where the *p* < 0.001.

## Results

### The Overview of the Analysis and Dataset

**FIGURE 1:**
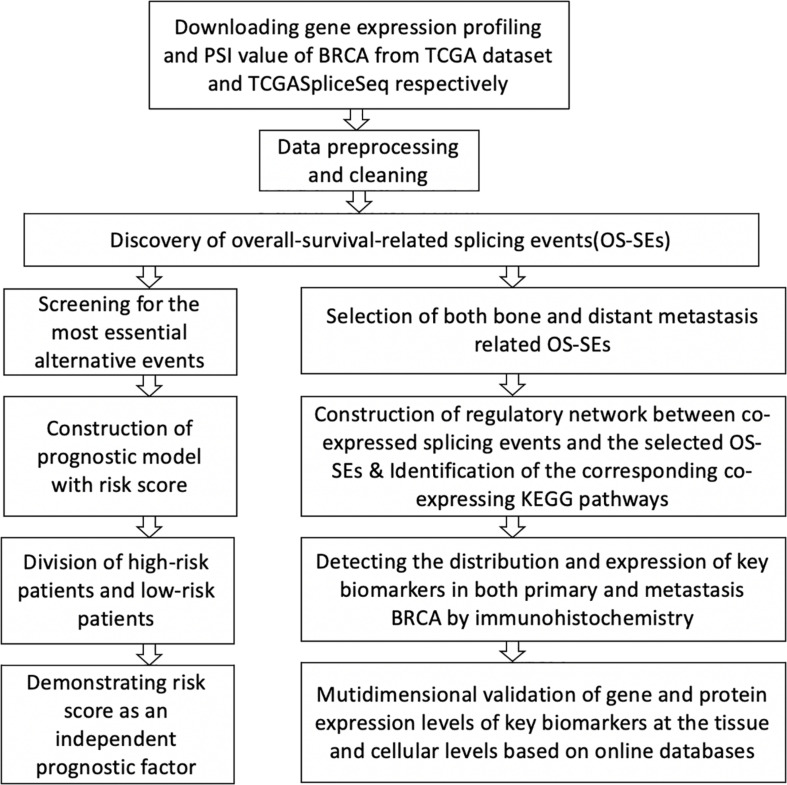
Schematic overview of the whole study.

The schematic view of this integrated analysis is depicted in Figure 1. Supplementary Table S1 summarizes the baseline characteristics of all patients. Patients were not one-to-one congruent with samples. The total amount of primary BRCA patients was 1,097, and the average survival time of the patients was 1,199 days with an average age of 58.5 years. In addition, there were only 1,080 primary samples; 32 of them underwent bone metastasis in the experimental group, and 1,048 samples did not experience bone metastasis in the control group.

**FIGURE 2:**
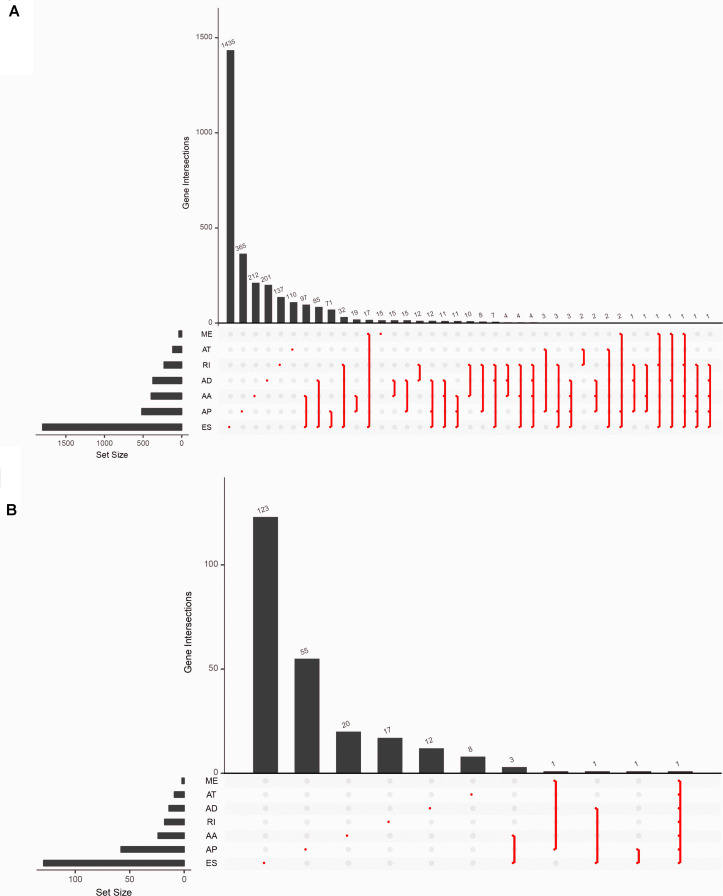
Overview of dataset. **(A)** The Upset plots of seven types of alternative splicing events and their parent genes. **(B)** The Upset plots of seven types of prognosis-related alternative splicing events and their parent genes. The lower part of each plot describes the permutations of the alternative splicing events; for each circumstance, the alternative splicing event is included if its corresponding place is filled with a red dot. The upper part of the plot represents the number of genes for each circumstance. AA, alternate acceptor; AD, alternate donor; AP, alternate promoter; AT, alternate terminator; ES, exon skip; ME, mutually exclusive exons; RI, retained intron.

There were 5,433 ASEs found in 2,933 parent genes in the samples with 2,728 ESs including 1,801 genes; 1,304 APs including 518 genes; 444 AAs including 396 genes; 414 ADs including 374 genes; 254 RIs including 229 genes; 251 ATs including 117 genes; and 38 MEs including 37 genes. One ASE could take place in different kinds of genes, and one gene could locate in different kinds of splicing patterns (Figure 2A and Supplementary Table S2). 294 of OS-ASEs were associated with survival status. Ranked by the magnitude of the association strength with survival, ES came first, followed by AP, AA, RI, and AD.

### Discoveries About Prognostic Model

**FIGURE 3:**
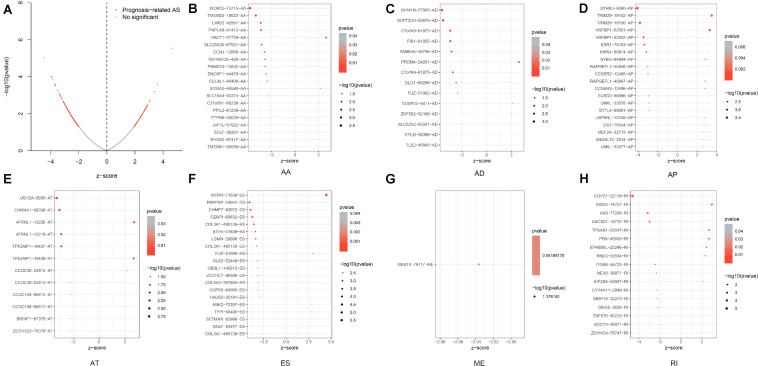
Discovery of prognosis-related ASEs and OS-SEs. **(A)** The volcano plot of the boundary between significant prognosis-related (red dots) and insignificant prognosis-related alternative splicing events (blue dots). **(B–H)** The Bubble plots of overall-survival-associated splicing events in each type of splicing patterns. AA, alternate acceptor; AD, alternate donor; AP, alternate promoter; AT, alternate terminator; ES, exon skip; ME, mutually exclusive exons; RI, retained intron.

Prognosis-related ASEs and their *P*-values were shown in the Upset plot and Volcano plot, respectively (Figures 2B, 3A). The Bubble plots illustrated the top 20 OS-SEs in seven types of splicing patterns (Figures 3B–H). Among all the ASEs, WDR55-73715-AA, KHNYN-27003-AD, DYRK3-9590-AP, USH2A-9805-AT, MTRR-71539-ES, GRB10-79717-ME, and COPZ1-22159-RI were the most significant events for AA, AD, AP, AT, ES, ME, and RI, respectively.

**FIGURE 4:**
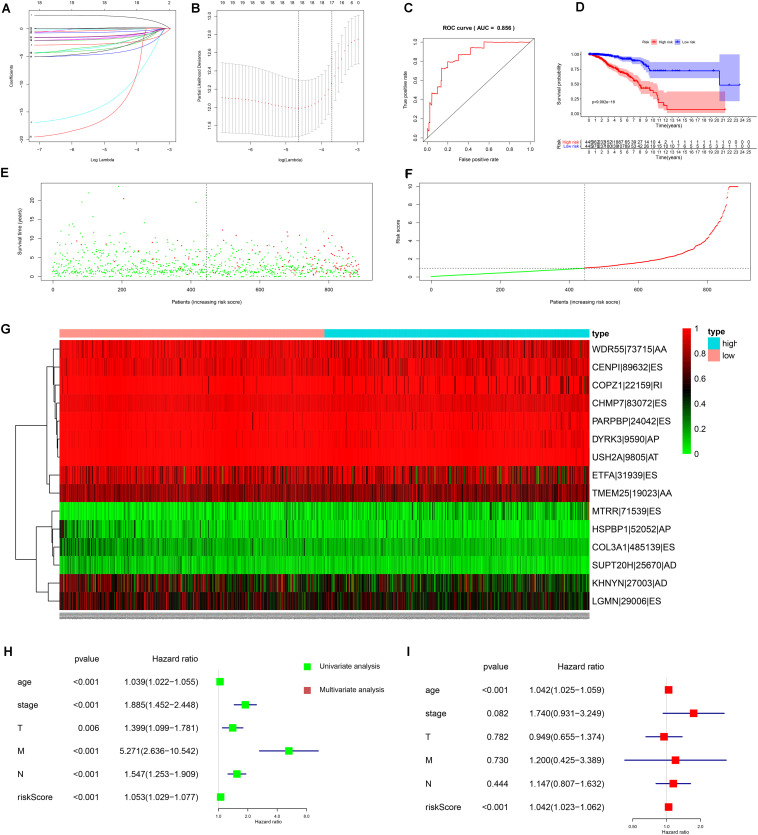
Construction and results of independent prognostic model. **(A)** The Lasso plot which determine of the number of overall-survival-associated splicing events in survival analysis by Lasso regression. **(B)** The Lambda plot which determine of the number of overall-survival-associated splicing events in survival analysis by Lasso regression. **(C)** The receiver operator characteristic curve (ROC) of survival analysis; the area under the ROC curve is 0.856. **(D)** The Kaplan–Meier plotter of the survival analysis in which low-risk patients (purple curve) is more likely to live longer than high-risks patients (red curve). **(E)** The scatterplot of survival time and the risk score of both low-risk patients (green points) and high-risk patients (red points). **(F)** The risk plot of both low-risk patients (green points) and high-risk patients (red points). **(G)** The heat map of 15 final-selected overall-survival-associated splicing events’ expression from the TCGA dataset. AA, alternate acceptor; AD, alternate donor; AP, alternate promoter; AT, alternate terminator; ES, exon skip; ME, mutually exclusive exons; RI, retained intron. **(H)** The forest plot of univariate Cox regression analysis. **(I)** The forest plot of multivariate Cox regression analysis.

Based on the top 20 OS-SEs, we built a prognostic model for BRCA. For avoiding over-fitting, Lasso plot (Figure 4A) and the Lambda plot (Figure 4B) were performed. Finally, 15 OS-SEs were selected to apply to the multivariate Cox regression analysis. The result of the regression was quite reliable with the ROC area under the curve (AUC) of 0.856 (Figure 4C). The median value of the risk score was 0.97, thus dividing the whole samples into two groups. The survival analysis results (Figure 4D) showed the survival probability of each group, indicating a vast difference between them, and the Kaplan–Meier curve demonstrates the reliability of the model by a *p* < 0.001. The risk curve and scatterplot indicated that samples with higher risk scores had a higher risk of mortality (Figures 4E,F). The heat map revealed that WDR55-73715-AA, CENPI-89632-ES, COPZ1-22159-RI, CHMP7-83072-ES, PARPBP-24042-ES, DYRK3-9590-AP, USH2A-9805-AT, ETFA-31939-ES, and TMEM25-19023-AA might have positive effects on BRCA while MTRR-71539-ES, HSPBP1-52052-AP, COL3A1-485139-ES, and SUPT20H-25670-AD may have adverse effects (Figure 4G).

In order to verify the independence of the risk score, both univariate and multivariate Cox regression analyses were applied to age, gender, stage, TNM stage, and risk score. With both *p*-values of the risk score in two analyses less than 0.001 and hazard ratios which were 1.053 [95% confidence interval (CI): 1.029–1.077] and 1.042 (95% CI: 1.023–1.062), the risk score proved to be a well-predicting model (Figures 4H,I). Consequently, SFs can serve as a predictor regarding survival.

### The Detection of the Potential Splicing Regulatory Network

There were 185 pathways detected by GSVA, which had potential prognostic predictive abilities. Then, just 26 pathways were selected as the prognosis-related pathways by univariate Cox analysis. Among them, ubiquitin-mediated proteolysis had the highest score of 3.36 (*P* = 0.027).

**FIGURE 5:**
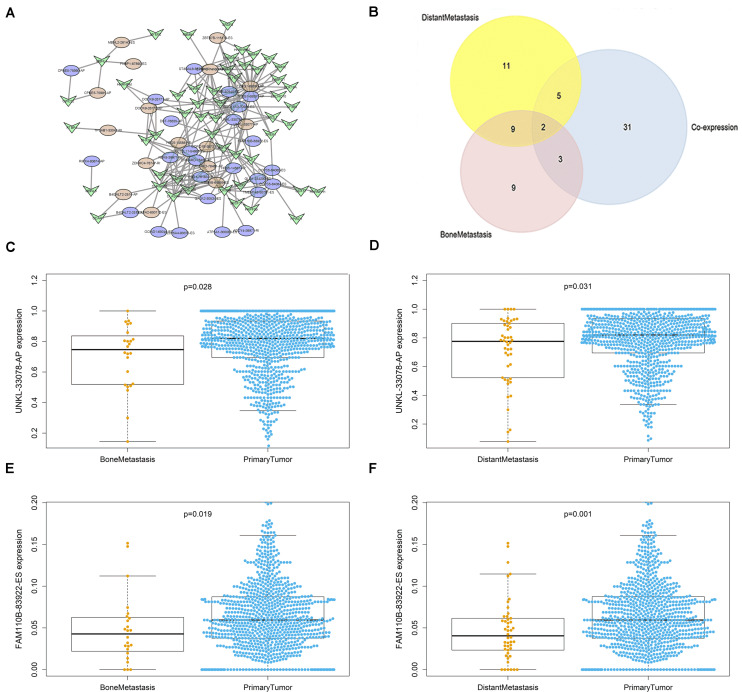
OS-SEs related to distant metastasis and bone metastasis. **(A)** The network of overall-survival-associated splicing events and their co-expressed splicing factors. **(B)** The Venn plot of overall-survival-associated splicing events related to distant metastasis and bone metastasis. **(C)** The Beeswarm plot of UNKL-33078-AP expression comparing both bone metastasis and primary tumor. **(D)** The Beeswarm plot of UNKL-33078-AP expression comparing both distant metastasis and primary tumor. **(E)** The Beeswarm plot of FAM110B-83922-ES expression comparing both bone metastasis and primary tumor. **(F)** The Beeswarm plot of FAM110B-83922-ES expression comparing both distant metastasis and primary tumor.

**FIGURE 6:**
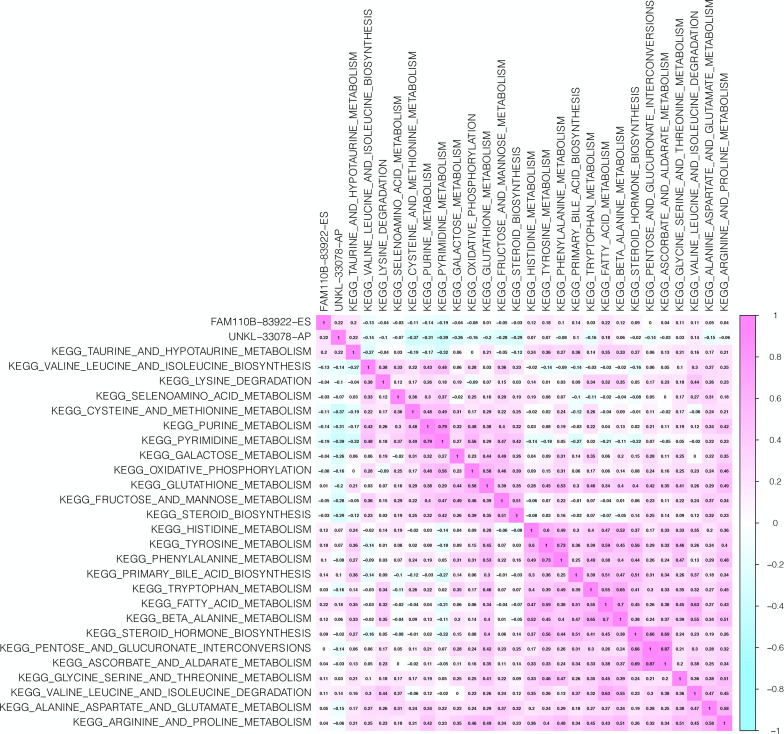
CorHeatmap of KEGG Pathways and Selected ASEs. The aim of this plot is to find out pathways correlated to overall-survival-associated splicing events associated with bone and distant metastasis.

The interacting and correlating relationship between SFs and OS-SEs was illustrated in the network (Figure 5A). During the detection of cancer status-related OS-SEs, 27, 23, and 43 OS-SEs were found related to distant metastasis, bone metastasis, and co-expression, respectively, and only 2 of them were collapsed (Figure 5B). The two OS-SEs in relation with both metastasis and stage were UNKL-33078-AP and FAM110B-83922-ES. The different expressions of these two OS-SEs between specific metastasis and primary tumor were depicted in boxplots (Figures 5C–F). The correlations between KEGG pathways and the two OS-SEs were showcased in the heat map (Figure 6) according to the result of Pearson analysis. Pyrimidine metabolism was the pathway most connected to UNKL (*R* = −0.385, *P* < 0.001), and fatty acid metabolism was the pathway most related to FAM110B (*R* = 0.223, *P* < 0.001).

### External Validation

The Human Protein Atlas (Supplementary Figure S1A) showed that FAM110B had a higher protein level in cells of BRCA than normal ones while UNKL was not detected in either normal or breast cancer. Thus, the study and conclusions were mainly focused on FAM110B and the related SF, CIRBP (*R* = 0.320, *P* < 0.001). The fatty acid metabolism, the most related KEGG pathway of FAM110B, revealed that ACAT2, ACAT1, ALOX15B, DHCR7, and ACAA1 were the five genes among the most centered genes (Supplementary Figure S11A). The Human Protein Atlas (Supplementary Figures S1B–F) demonstrated that all of these five genes have a relatively high level of protein expression.

The expression levels of CIRBP, FAM110B, ACAT1, ACAT2, ACAA1, ALOX15B, and DHCR7 from online databases are summarized in Supplementary Table S3. GTEx (Supplementary Figure S2) revealed that ACAT2 was lowly expressed in normal thyroid. ACAT1 and ACAT2 were lowly expressed, while FAM110B, ALOX15B, and DHCR7 were highly expressed at the tissue level in BRCA Oncomine (Supplementary Figure S9). CCLE (Supplementary Figure S10) demonstrated that FAM110B and ALOX15B were lowly expressed, while CIRBP, ACAT1, ACAT2, ACAA1, and DHCR7 were highly expressed at the tissue level in BRCA.

Then, a survival analysis of each gene was carried out using different databases, and the results are summarized in Supplementary Table S4. PROGgeneV2 (Supplementary Figure S3) demonstrated that FAM110B (*P* = 0.043), CIRBP (*P* < 0.001), ACAT2 (*P* < 0.001), ACAT1 (*P* = 0.003), ALOX15B (*P* = 0.010), DHCR7 (*P* < 0.001), and ACAA1 (*P* < 0.001) were independent prognostic factors. GEPIA (Supplementary Figure S4) showed that CIRBP was related to survival (*P* = 0.010). The Kaplan–Meier plotter (Supplementary Figure S5) illustrated that FAM110B (*P* < 0.001), CIRBP (*P* < 0.001), ACAT2 (*P* < 0.001), ACAT1 (*P* < 0.001), ALOX15B (*P* < 0.001), DHCR7 (*P* < 0.001), and ACAA1 (*P* = 0.019) were all shown to be prognostic indicators. UALCAN (Supplementary Figure S6) manifested that FAM110B (*P* = 0.004), CIRBP (*P* = 0.008), ACAT2 (*P* = 0.002), ACAT1 (*P* = 0.020), ALOX15B (*P* = 0.029), DHCR7 (*P* = 0.013), and ACAA1 (*P* = 0.026) were all prognostic indicators. Linkedomics (Supplementary Figure S7) revealed that ACAT1 (*P* = 0.193), ALOX15B (*P* = 0.016), and ACAA1 (*P* = 0.014) were related to overall survival of prognosis. cBioportal (Supplementary Figure S8) demonstrated that FAM110B (*P* < 0.001), CIRBP (*P* = 0.045), ACAT1 (*P* < 0.001), and DHCR7 (*P* = 0.003) were significantly related to prognosis.

**FIGURE 7:**
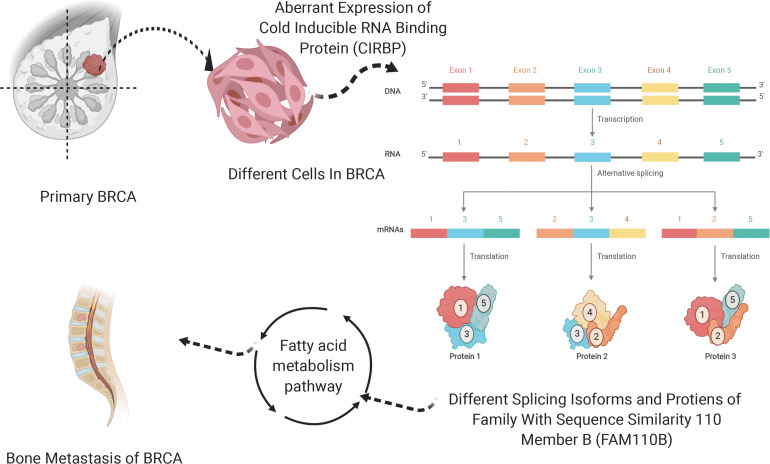
Schematic diagram of the scientific hypothesis. OS-SEs (FAM110B-83922-ES) regulates SF (CIRBP) by the downstream pathway (Fatty acid metabolism pathway).

In the end, the interaction PPI network of FAM110B, CIRBP, ACAT2, ACAT1, ALOX15B, DHCR7, and ACAA1 from String (Supplementary Figure S11B) was shown. Thus, we hypothesized that aberrant CIRBP regulated FAM110B-83922-ES in ties with the tumorigenesis, metastasis, and prognosis of BRCA via fatty acid metabolism, and the schematic diagram of this scientific hypothesis is depicted in Figure 7.

### Independent Dataset Validation and Spatial Transcriptome Validation

**FIGURE 8:**
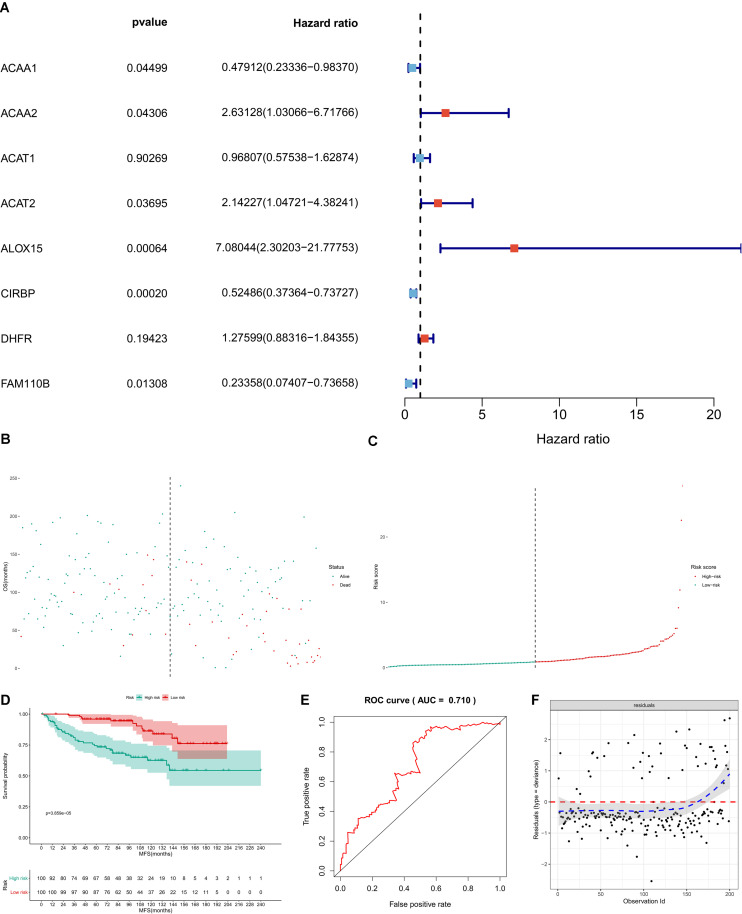
Independent dataset validation by GSE11121. In GSE11121, univariate Cox regression analysis suggested that CIRBP (*P* < 0.001), FAM110B (*P* = 0.013), ACAA1 (*P* = 0.045), ACAA2 (*P* = 0.043), ACAT2 (*P* = 0.037), and ALOX15 (*P* < 0.001) had a significant association with metastasis-free survival (MFS) of BRCA patients **(A)**. Then, these key genes were incorporated into the multivariate Cox model, and the risk score for each BRCA patient was calculated by the formula of the multivariate model. The risk line and scatterplot illustrated the distribution of risk score among all patients **(B,C)**. The Kaplan–Meier survival curve showed the significantly prognostic value of the risk score (**D**, *P* < 0.001). Furthermore, the accuracy and goodness of fit (GOF) of the multivariate Cox regression model were illustrated by the ROC curve (AUC = 0.710) and the residual plot **(E,F)**.

In GSE11121, univariate Cox regression analysis suggested that CIRBP (*P* < 0.001), FAM110B (*P* = 0.013), ACAA1 (*P* = 0.045), ACAA2 (*P* = 0.043), ACAT2 (*P* = 0.037), and ALOX15 (*P* < 0.001) had a significant association with metastasis-free survival (MFS) of BRCA patients (Figure 8A). Then, these key genes were incorporated into the multivariate Cox model and the risk score for each BRCA patient was calculated by the formula of the multivariate model. The risk line and scatterplot illustrated the distribution of the risk score among all patients (Figures 8B,C). The Kaplan–Meier survival curve showed the significantly prognostic value of the risk score (Figure 8D, *P* < 0.001). Furthermore, the accuracy and goodness of fit (GOF) of the multivariate Cox regression model was illustrated by the ROC curve (AUC = 0.710) and the residual plot (Figures 8E,F).

**FIGURE 9:**
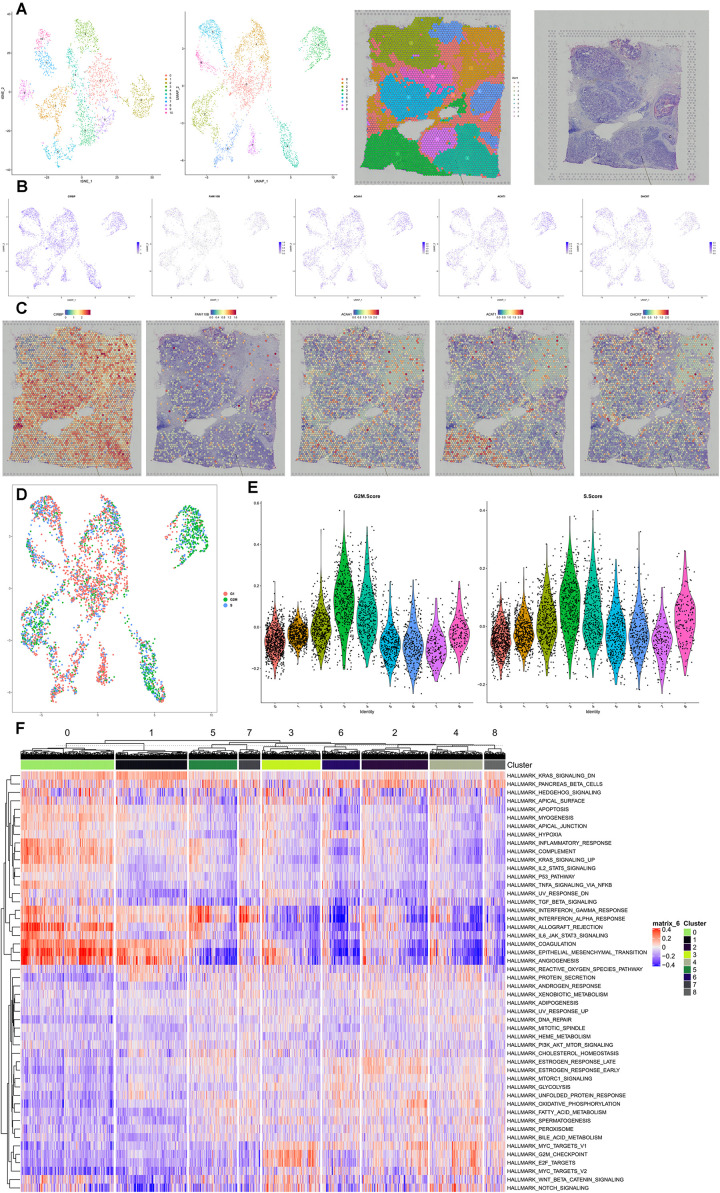
Spatial transcriptome validation. Spatial transcriptome combined with scRNA-seq data of BRCA was used to identify the cell subtype localization of the key genes in the speculative regulatory mechanism. Eleven clusters and nine clusters were identified by t-SNE and UMAP **(A)**. Invasive ductal carcinoma and intraductal carcinoma *in situ* as well as normal tissue were clearly demonstrated in HE staining slides **(A)**. The feature plots and spatial feature plots were utilized to illustrate the distribution and expression of CIRBP, FAM110B, ACAA1, ACAT1, and DHCR7, showing that these key genes were highly expressed in the invasive ductal carcinoma tissue (clusters 2, 5, 8) **(B,C)**. Additionally, the cell-cycle analysis suggested that most cells highly expressing these key genes were in the phase of G2M and S **(D,E)**, and those cell-division-related signaling pathways such as HALLMARK_G2M_CHECKPOINT and HALLMARK_E2F_TARGETS were activated while some tumor-suppressor signaling pathways such as HALLMARK_P53_PATHWAY and HALLMARK_TNFA_SIGNALING_VIA_NFKB were downregulated in the invasive ductal carcinoma tissue **(F)**.

Spatial transcriptome combined with scRNA-seq data of BRCA was used to identify the cell subtype localization of the key genes in the speculative regulatory mechanism. Eleven clusters and nine clusters were identified by t-SNE and UMAP, respectively (Figure 9A). Invasive ductal carcinoma and intraductal carcinoma *in situ* as well as normal tissue were clearly demonstrated in HE staining slides (Figure 9A). The feature plots and spatial feature plots were utilized to illustrate the distribution and expression of CIRBP, FAM110B, ACAA1, ACAT1, and DHCR7, showing that these key genes were highly expressed in the invasive ductal carcinoma tissue (cluster 2, 5, 8) (Figures 9B,C). Additionally, the cell-cycle analysis suggested that most cells highly expressing these key genes were in the phase of G2M and S (Figures 9D,E), and those cell-division-related signaling pathways such as HALLMARK_G2M_CHECKPOINT and HALLMARK_E2F_TARGETS were activated while some tumor-suppressor signaling pathways such as HALLMARK_ P53_PATHWAY and HALLMARK_TNFA_SIGNALING_VIA_ NFKB were downregulated in the invasive ductal carcinoma tissue (Figure 9F).

## Discussion

BRCA is women’s most common neoplasm, whose 5-year survival rate is less than 40% in some areas of the world (Akram et al., 2017). The incidence and mortality rates of BRCA are still expected to increase in the next 5–10 years (Anastasiadi et al., 2017). BRCA could be spread to sites like bone, lungs, the liver, and axillary lymph nodes (Harbeck et al., 2019); death is more likely to occur due to metastasis (Akram et al., 2017). Therefore, it is quite essential for us to delve into BRCA wishing for an improvement of the diagnosis and treatment. ASEs frequently happened in eukaryote lineages (Bush et al., 2017), and alternatively spliced isoforms are generated in more than ninety-five percent of multi-exon genes in human beings (Pan et al., 2008; Wang et al., 2008). In addition, ASEs along with SFs are considered to have essential impacts on the development and progression of tumors (Srebrow and Kornblihtt, 2006), and AS changes might become independent oncogenic processes (Climente-Gonzalez et al., 2017). Most previous studies of BRCA are focused on alterations at the transcriptome level, but analysis of the posttranscriptional process is largely ignored. One study found that some ASEs in gene NF-YA are associated with BRCA (Dolfini et al., 2019). Another study also focused on some OS-SEs in BRCA and analyzed some genes associated with OS-SEs (Zhang D. et al., 2019). Despite the significance, metastasis-related ASEs and potential therapeutic targets were ignored, and the relationship between SF and ASEs was also not analyzed.

In this study, univariate Cox regression and Lasso regression selected 15 OS-SEs and a prognostic model was established showing that SFs could well predict survival. Two OS-SEs were identified to be associated with tumorigenesis and metastasis, and their related SFs were selected along with corresponding prognostic KEGG pathways. After multidimensional validation, we finally assumed that CIRBP regulated FAM110B-83922-ES of BRCA via fatty acid metabolism, and five proteins involved in fatty acid metabolism were demonstrated to be highly related to survival as well. The prognostic model could be an effectual tool for doctors in the future, and the assumption might provide an idea for finding a particular target to increase the survival rate of BRCA.

CIRBP, cold-inducible RNA-binding protein, belonging to the cold-shock protein family, is expressed in different cell types and was involved in various cancer pathological processes, including cell survival, cell proliferation, and cancer (Zhu et al., 2016; Su et al., 2020). It was reported that CIRBP had different effects in various cancers according to the cell context such as acting as a tumor suppressor in rectal carcinoma, endometrial carcinoma, and ovarian tumor and having pro-tumorigenic influences on melanoma, colorectal cancer, prostate cancer, central-nervous-system-related tumor, liver-pancreas carcinomas, skin squamous cell carcinoma, bladder cancer, and pituitary corticotrope adenoma (Zhu et al., 2016; Liao et al., 2017; Zhong and Huang, 2017; Chen et al., 2018; Colasanti et al., 2018; Lujan et al., 2018; Lin et al., 2019). Some studies found down-expressed CIRBP to be always related with poor-survival cell-cycle process and disease stage in BRCA patients (Joe and Nam, 2016; Rodrigues-Peres et al., 2019). Additionally, a protein microarray experimental study sheds light on CIRBP as an autoantibody target potentially used for early BRCA detection (Lacombe et al., 2014). Studies also identified CIRBP as a potential prognosis-related gene which could be used in BRCA prognostic prediction (Shimizu and Nakayama, 2019). Acting as a cold-inducible RNA-binding protein, CIRBP also had a positive influence on HuR protein levels concerned with BRCA formation (Kotta-Loizou et al., 2016). A study also found the co-regulated expression of SFs in BRCA, indicating that CIRBP expressed much lower in tumor tissue than in metastatic tissue (Koedoot et al., 2019).

FAM110B, family with sequence similarity 110 member B, localized in centrosomes, and the progress in the G1 phase of the cell cycle is affected by the ectopic expression of its protein (Hauge et al., 2007). Much of the researches focused on the relationship between FAM110B and BRCA. FAM110B played a negative role in the translational regulation of BRCA1, which is a tumor suppressor of BRCA (Dacheux et al., 2013). Previous studies have also demonstrated that the signature of FAM110B along with 40 other genes can serve as a survival predictor for BRCA. The signature was associated not only with age and ER status but also with overall survival and distant metastasis-free survival (Yin et al., 2014). What is more, FAM110B expressed significantly lower in young women compared to older women with BRCA, so that it may play quite different roles in the development of BRCA in young women than in older women (Colak et al., 2013).

Until now, there is no evidence about the direct regulation between CIRBP and FAM110B. Furthermore, they are both involved in cell-cycle progression based on the up-to-date reports (Hauge et al., 2007; Zhu et al., 2016; Su et al., 2020), and we found out that they are co-expressed in the BRCA database. To go through the mechanism of CIRBP regulating FAM110B-83922-ES, fatty acid metabolism was detected as the possible pathway connecting them.

Through divergent mechanisms, endogenous fatty acid status could have a great impact on health and disease (Brenna et al., 2018). Consistent with our study, many studies illustrated the significance of fatty acid metabolism in BRCA. Characteristics of different types of BRCA vary in fatty acid metabolism (Yamashita et al., 2017). In addition, fatty acid metabolism was engaged in BRCA progression, and some proteins related to fatty acid transport revealed to enhance the possibility of migration and invasion of BRCA (Yen et al., 2018). Furthermore, evidence demonstrated that fatty acid metabolism was considered a potential target for BRCA therapy (Kinlaw et al., 2016).

In sum, although the results of our study were quite reliable, our study still had some limitations. The result was only based on one single chart of sequencing data and computer analyses where the sample size of the dataset and the accuracy of molecular mechanisms were limited. However, the most important shortcoming is that systematic errors could not be avoided and wet experiments and clinical trials are required to perform to verify the results. The inherent vice of *in silico* analysis was bias based on different platforms, and our scientific hypothesis only based on bioinformation instead of mechanism exploring. In addition, a small sample size for bone metastasis samples may result in an unbalance of the datasets. In order to better diminish the bias, external multidimensional validation was followed to minimize the negative impact of this limitation, showing the reliability of our findings.

## Conclusion

In this comparative bioinformatics analysis, we constructed a prognostic model of BRCA. In addition, our further findings suggest that the aberrant splicing factor, CIRBP, regulated an alternative splicing event, the exon skip of FAM110B, during which the fatty acid metabolism pathway might play an essential part in tumorigenesis and prognosis of BRCA. This scientific proposition might provide direct instruction for the following biological experiments.

## Data Availability Statement

Publicly available datasets were analyzed in this study. This data can be found here: the code and datasets generated for this study are included in the Supplementary Material, TCGA-BRCA program (https://portal.gdc.cancer.gov), GSE11121 (https://www.ncbi.nlm.nih.gov/geo/query/acc.cgi?acc=GSE11121) and 10xgenomics dataset (https://support.10xgenomics.com/spatial-gene-expression/datasets/1.1.0/V1_Breast_Cancer_Block_A_Section_1).

## Ethics Statement

This study was approved by the Ethics Committee of the First Affiliated Hospital of Zhengzhou University. The patients/participants provided their written informed consent to participate in this study.

## Author Contributions

RH, JG, PY, SZ, PH, XZ, JiaZ, YQ, YZ, HL, LH, JieZ, DY, and ZH: conception and design, collection and assembly of data, data analysis and interpretation, and final approval of manuscript. RH, JG, PY, JieZ, DY, and ZH: manuscript writing. All authors contributed to the article and approved the submitted version.

## Conflict of Interest

The authors declare that the research was conducted in the absence of any commercial or financial relationships that could be construed as a potential conflict of interest.

## Footnotes

1 https://portal.gdc.cancer.gov/

2 https://bioinformatics.mdanderson.org/TCGASpliceSeq/

3 https://pathcards.genecards.org/

4 https://www.ncbi.nlm.nih.gov/geo/query/acc.cgi?acc=GSE11121

5 https://support.10xgenomics.com/spatial-gene-expression/datasets/1.1.0/V1_Breast_Cancer_Block_A_Section_1

6 https://www.gsea-msigdb.org/gsea/msigdb/index.jsp

7 www.r-project.org

## Supplementary Material

FIGURE S1 External validation of FAM110B **(A)**, ACAT1 **(B)**, ACAT2 **(C)**, ACAA1 **(D)**, ALOX15B **(E)**, and DHCR7 **(F)** by the Human Protein Atlas.

FIGURE S2 External validation of CIRBP **(A)**, FAM110B **(B)**, ACAT1 **(C)**, ACAT2 **(D)**, ACAA1 **(E)**, ALOX15B **(F)**, and DHCR7 (G) by GTEx.

FIGURE S3 External validation of CIRBP **(A)**, FAM110B **(B)**, ACAT1 **(C)**, ACAT2 **(D)**, ACAA1 **(E)**, ALOX15B **(F)**, and DHCR7 (G) by PROGgeneV2. CIRBP, FAM110B, ACAT1, and ACAA1 high-expressed in tissue level in BRCA; while ACAT2 and DHCR7 low-expressed in tissue level in BRCA.

FIGURE S4 External validation of CIRBP **(A)**, FAM110B **(B)**, ACAT1 **(C)**, ACAT2 **(D)**, ACAA1 **(E)**, ALOX15B **(F)**, and DHCR7 **(G)** by GEPIA.

FIGURE S5 External validation of CIRBP **(A)**, FAM110B **(B)**, ACAT1 **(C)**, ACAT2 **(D)**, ACAA1 **(E)**, ALOX15B **(F)**, and DHCR7 **(G)** by The Kaplan Meier plotter. CIRBP, FAM110B, ACAT1, ALOX15B, and ACAA1 high-expressed in tissue level in BRCA; while ACAT2 and DHCR7 low-expressed in tissue level in BRCA.

FIGURE S6 External validation of CIRBP **(A)**, FAM110B **(B)**, ACAT1 **(C)**, ACAT2 **(D)**, ACAA1 **(E)**, ALOX15B **(F)**, and DHCR7 **(G)** by UALCAN. ALOX15B expressed lowly in BRCA; CIRBP, FAM110B, ACAT1, ACAT2, ACAA1, and DHCR7 expressed highly in normal thyroid and BRCA.

FIGURE S7 External validation of CIRBP **(A)**, FAM110B **(B)**, ACAT1 **(C)**, ACAT2 **(D)**, ACAA1 **(E)**, ALOX15B **(F)**, and DHCR7 **(G)** by Linkedomics.

FIGURE S8 External validation of CIRBP **(A)**, FAM110B **(B)**, ACAT1 **(C)**, ACAT2 **(D)**, ACAA1 **(E)**, ALOX15B **(F)**, and DHCR7 **(G)** by cBioportal.

FIGURE S9 External validation of CIRBP **(A)**, FAM110B **(B)**, ACAT1 **(C)**, ACAT2 **(D)**, ACAA1 **(E)**, ALOX15B **(F)**, and DHCR7 **(G)** by Oncomine. ACAT1 and ACAT2 low-expressed, while FAM110B, ALOX15B, and DHCR7 high-expressed in tissue level in BRCA.

FIGURE S10 External validation of CIRBP **(A)**, FAM110B **(B)**, ACAT1 **(C)**, ACAT2 **(D)**, ACAA1 **(E)**, ALOX15B **(F)**, and DHCR7 **(G)** by CCLE. FAM110B and ALOX15B low-expressed, while CIRBP, ACAT1, ACAT2, ACAA1, and DHCR7 high-expressed in cellular level in BRCA.

FIGURE S11 The protein-protein interaction networks. **(A)** The protein network of fatty acid metabolism pathway from Pathcards. **(B)** The protein network of CIRBP, FAM110B, ACAT1, ACAT2, ACAA1, ALOX15B, and DHCR7 from String.

TABLE S1 Description of patients’ dataset.

TABLE S2 Number of ASEs and number of ASEs in different ASE type.

TABLE S3 The external validation of CIRBP, FAM110B, ACAT1, ACAT2, ACAA1, ALOX15B, and DHCR7.

TABLE S4 Summary of external validation.

## Abbreviations

AA: alternate acceptor
AD: alternate donor
AP: alternate promoter
AS: alternative splicing
ASEs: alternative splicing events
AT: alternate terminator
AUC: area under the ROC curve
BRCA: breast cancer
ES: exon skip
GEPIA: Gene Expression Profiling Interactive Analysis
GO: Gene Ontology
GSVA: Gene Set Variation Analysis
KEGG: Kyoto Encyclopedia of Genes and Genomes
M: metastasis
ME: mutually exclusive exons
N: regional lymph node
OS: overall survival
PI: prognostic index
RI: retained intron
ROC: receiver operating characteristic curve
SE: splicing events
SF: splicing factor
TCGA: The Cancer Genome Atlas
TNM stage: T: tumor.

## References

[B1] Akram M. Iqbal M. Daniyal M. Khan A. U. (2017). Awareness and current knowledge of breast cancer. *Biol. Res.* 50:33. 10.1186/s40659-017-0140-9 PMC5625777 28969709

[B2] Anastasiadi Z. Lianos G. D. Ignatiadou E. Harissis H. V. Mitsis M. (2017). Breast cancer in young women: an overview. *Updates Surg.* 69 313–317. 28260181 10.1007/s13304-017-0424-1

[B3] Aran D. Looney A. P. Liu L. Wu E. Fong V. Hsu A. (2019). Reference-based analysis of lung single-cell sequencing reveals a transitional profibrotic macrophage. *Nat. Immunol.* 20 163–172. 10.1038/s41590-018-0276-y 30643263 PMC6340744

[B4] Brenna J. T. Plourde M. Stark K. D. Jones P. J. Lin Y. H. (2018). Best practices for the design, laboratory analysis, and reporting of trials involving fatty acids. *Am. J. Clin. Nutr.* 108 211–227. 10.1093/ajcn/nqy089 29931035 PMC6084616

[B5] Bush S. J. Chen L. Tovar-Corona J. M. Urrutia A. O. (2017). Alternative splicing and the evolution of phenotypic novelty. *Philos. Trans. R. Soc. Lond. B Biol. Sci.* 372:20150474. 10.1098/rstb.2015.0474 27994117 PMC5182408

[B6] Butler A. Hoffman P. Smibert P. Papalexi E. Satija R. (2018). Integrating single-cell transcriptomic data across different conditions, technologies, and species. *Nat. Biotechnol.* 36 411–420. 10.1038/nbt.4096 29608179 PMC6700744

[B7] Cardoso F. Senkus E. Costa A. Papadopoulos E. Aapro M. Andre F. (2018). 4th ESO-ESMO international consensus guidelines for advanced breast cancer (ABC 4)dagger. *Ann. Oncol.* 29 1634–1657. 30032243 10.1093/annonc/mdy192 PMC7360146

[B8] Cerami E. Gao J. Dogrusoz U. Gross B. E. Sumer S. O. Aksoy B. A. (2012). The cBio cancer genomics portal: an open platform for exploring multidimensional cancer genomics data. *Cancer Discovery* 2 401–404. 10.1158/2159-8290.cd-12-0095 22588877 PMC3956037

[B9] Chandrashekar D. S. Bashel B. Balasubramanya S. A. H. Creighton C. J. Ponce-Rodriguez I. Chakravarthi B. (2017). UALCAN: a portal for facilitating tumor subgroup gene expression and survival analyses. *Neoplasia* 19 649–658. 10.1016/j.neo.2017.05.002 28732212 PMC5516091

[B10] Chen J. K. Lin W. L. Chen Z. Liu H. W. (2018). PARP-1-dependent recruitment of cold-inducible RNA-binding protein promotes double-strand break repair and genome stability. *Proc. Natl. Acad. Sci. U.S.A.* 115 E1759–E1768. 29432179 10.1073/pnas.1713912115 PMC5828585

[B11] Chung N. C. Storey J. D. (2015). Statistical significance of variables driving systematic variation in high-dimensional data. *Bioinformatics* 31 545–554. 10.1093/bioinformatics/btu674 25336500 PMC4325543

[B12] Climente-Gonzalez H. Porta-Pardo E. Godzik A. Eyras E. (2017). The functional impact of alternative splicing in cancer. *Cell Rep.* 20 2215–2226. 10.1016/j.celrep.2017.08.012 28854369

[B13] Colak D. Nofal A. Albakheet A. Nirmal M. Jeprel H. Eldali A. (2013). Age-specific gene expression signatures for breast tumors and cross-species conserved potential cancer progression markers in young women. *PLoS One* 8:e63204. 10.1371/journal.pone.0063204 23704896 PMC3660335

[B14] Colasanti T. Fiorito S. Alessandri C. Serafino A. Andreola F. Barbati C. (2018). Diesel exhaust particles induce autophagy and citrullination in normal human bronchial epithelial cells. *Cell Death Dis.* 9:1073. 10.1038/s41419-018-1111-y PMC6195610 30341285

[B15] Consortium G. (2015). Human genomics. the genotype-tissue expression (GTEx) pilot analysis: multitissue gene regulation in humans. *Science* 348 648–660. 25954001 10.1126/science.1262110 PMC4547484

[B16] Dacheux E. Vincent A. Nazaret N. Combet C. Wierinckx A. Mazoyer S. (2013). BRCA1-dependent translational regulation in breast cancer cells. *PLoS One* 8:e67313. 10.1371/journal.pone.0067313 23805307 PMC3689694

[B17] Dolfini D. Andrioletti V. Mantovani R. (2019). Overexpression and alternative splicing of NF-YA in breast cancer. *Sci. Rep.* 9:12955. 10.1038/s41598-019-49297-5 PMC6736888 31506469

[B18] Early Breast Cancer Trialists’ Collaborative Group Peto R. Davies C. Godwin J. Gray R. Pan H. C. (2012). Comparisons between different polychemotherapy regimens for early breast cancer: meta-analyses of long-term outcome among 100,000 women in 123 randomised trials. *Lancet* 379 432–444. 10.1016/s0140-6736(11)61625-5 22152853 PMC3273723

[B19] Early Breast Cancer Trialists’ Collaborative Group Davies C. Godwin J. Gray R. Clarke M. Cutter D. (2011). Relevance of breast cancer hormone receptors and other factors to the efficacy of adjuvant tamoxifen: patient-level meta-analysis of randomised trials. *Lancet* 378 771–784. 10.1016/s0140-6736(11)60993-8 21802721 PMC3163848

[B20] Ghandi M. Huang F. W. Jane-Valbuena J. Kryukov G. V. Lo C. C. Mcdonald E. R. (2019). Next-generation characterization of the cancer cell line encyclopedia. *Nature* 569 503–508. 31068700 10.1038/s41586-019-1186-3 PMC6697103

[B21] Goswami C. P. Nakshatri H. (2014). PROGgeneV2: enhancements on the existing database. *BMC Cancer* 14:970. 10.1186/1471-2407-14-970 25518851 PMC4300843

[B22] Hanzelmann S. Castelo R. Guinney J. (2013). GSVA: gene set variation analysis for microarray and RNA-seq data. *BMC Bioinformatics* 14:7. 10.1186/1471-2105-14-7 23323831 PMC3618321

[B23] Harbeck N. Penault-Llorca F. Cortes J. Gnant M. Houssami N. Poortmans P. (2019). Breast cancer. *Nat. Rev. Dis. Primers* 5:66. 10.1038/s41572-019-0111-2 31548545

[B24] Hauge H. Patzke S. Aasheim H. C. (2007). Characterization of the FAM110 gene family. *Genomics* 90 14–27. 10.1016/j.ygeno.2007.03.002 17499476

[B25] Hou R. Denisenko E. Forrest A. R. R. (2019). scMatch: a single-cell gene expression profile annotation tool using reference datasets. *Bioinformatics* 35 4688–4695. 10.1093/bioinformatics/btz292 31028376 PMC6853649

[B26] Joe S. Nam H. (2016). Prognostic factor analysis for breast cancer using gene expression profiles. *BMC Med. Inform. Decis. Mak.* 16 (Suppl. 1):56. 10.1186/s12911-016-0292-5 27454576 PMC4959370

[B27] Kinlaw W. B. Baures P. W. Lupien L. E. Davis W. L. Kuemmerle N. B. (2016). Fatty acids and breast cancer: make them on site or have them delivered. *J. Cell Physiol.* 231 2128–2141. 10.1002/jcp.25332 26844415 PMC4912394

[B28] Koedoot E. Smid M. Foekens J. A. Martens J. W. M. Le Devedec S. E. Van De Water B. (2019). Co-regulated gene expression of splicing factors as drivers of cancer progression. *Sci Rep* 9;5484. 10.1038/s41598-019-40759-4 PMC6445126 30940821

[B29] Kotta-Loizou I. Vasilopoulos S. N. Coutts R. H. Theocharis S. (2016). Current evidence and future perspectives on hur and breast cancer development, prognosis, and treatment. *Neoplasia* 18 674–688. 10.1016/j.neo.2016.09.002 27764700 PMC5071540

[B30] Kouyama Y. Masuda T. Fujii A. Ogawa Y. Sato K. Tobo T. (2019). Oncogenic splicing abnormalities induced by DEAD-Box Helicase 56 amplification in colorectal cancer. *Cancer Sci.* 110 3132–3144. 10.1111/cas.14163 31390121 PMC6778637

[B31] Lacombe J. Mange A. Solassol J. (2014). Use of autoantibodies to detect the onset of breast cancer. *J. Immunol. Res.* 2014:574981. 10.1155/2014/574981 PMC4131063 25143958

[B32] Lee S. C. Abdel-Wahab O. (2016). Therapeutic targeting of splicing in cancer. *Nat. Med.* 22 976–986. 10.1038/nm.4165 27603132 PMC5644489

[B33] Liao Y. Tong L. Tang L. Wu S. (2017). The role of cold-inducible RNA binding protein in cell stress response. *Int. J. Cancer* 141 2164–2173. 10.1002/ijc.30833 28608439

[B34] Liberzon A. Birger C. Thorvaldsdóttir H. Ghandi M. Mesirov J. P. Tamayo P. (2015). The molecular signatures database (MSigDB) hallmark gene set collection. *Cell Syst.* 1 417–425. 10.1016/j.cels.2015.12.004 26771021 PMC4707969

[B35] Lin T. Y. Chen Y. Jia J. S. Zhou C. Lian M. Wen Y. T. (2019). Loss of Cirbp expression is correlated with the malignant progression and poor prognosis in nasopharyngeal carcinoma. *Cancer Manag. Res.* 11 6959–6969. 10.2147/cmar.s211389 31413636 PMC6662521

[B36] Lujan D. A. Ochoa J. L. Hartley R. S. (2018). Cold-inducible RNA binding protein in cancer and inflammation. *Wiley Interdiscip. Rev. RNA* 9; 10.1002/wrna.1462 . 29322631 PMC5886743

[B37] Mclaughlin S. A. (2013). Surgical management of the breast: breast conservation therapy and mastectomy. *Surg. Clin. North Am.* 93 411–428. 23464693 10.1016/j.suc.2012.12.006

[B38] Nagy A. Lanczky A. Menyhart O. Gyorffy B. (2018). Validation of miRNA prognostic power in hepatocellular carcinoma using expression data of independent datasets. *Sci. Rep.* 8;9227. 10.1038/s41598-018-27521-y PMC6003936 29907753

[B39] Pan Q. Shai O. Lee L. J. Frey B. J. Blencowe B. J. (2008). Deep surveying of alternative splicing complexity in the human transcriptome by high-throughput sequencing. *Nat. Genet.* 40 1413–1415. 10.1038/ng.259 18978789

[B40] Piva F. Giulietti M. Nocchi L. Principato G. (2009). SpliceAid: a database of experimental RNA target motifs bound by splicing proteins in humans. *Bioinformatics* 25 1211–1213. 10.1093/bioinformatics/btp124 19261717

[B41] Rhodes D. R. Yu J. Shanker K. Deshpande N. Varambally R. Ghosh D. (2004). ONCOMINE: a cancer microarray database and integrated data-mining platform. *Neoplasia* 6 1–6. 10.1016/s1476-5586(04)80047-2 15068665 PMC1635162

[B42] Rodrigues-Peres R. M. De S. C. B. Anurag M. Lei J. T. Conz L. Goncalves R. (2019). Copy number alterations associated with clinical features in an underrepresented population with breast cancer. *Mol. Genet. Genomic. Med.* 7:e00750. 10.1002/mgg3.750 PMC6625096 31099189

[B43] Ryan M. Wong W. C. Brown R. Akbani R. Su X. Broom B. (2016). TCGASpliceSeq a compendium of alternative mRNA splicing in cancer. *Nucleic Acids Res.* 44 D1018–D1022. 26602693 10.1093/nar/gkv1288 PMC4702910

[B44] Schmidt M. Böhm D. Von Törne C. Steiner E. Puhl A. Pilch H. (2008). The humoral immune system has a key prognostic impact in node-negative breast cancer. *Cancer Res.* 68 5405–5413. 10.1158/0008-5472.can-07-5206 18593943

[B45] Shannon P. Markiel A. Ozier O. Baliga N. S. Wang J. T. Ramage D. (2003). Cytoscape: a software environment for integrated models of biomolecular interaction networks. *Genome Res.* 13 2498–2504. 10.1101/gr.1239303 14597658 PMC403769

[B46] Shimizu H. Nakayama K. I. (2019). A 23 gene-based molecular prognostic score precisely predicts overall survival of breast cancer patients. *EBioMedicine* 46 150–159. 10.1016/j.ebiom.2019.07.046 31358476 PMC6711850

[B47] Snel B. Lehmann G. Bork P. Huynen M. A. (2000). STRING: a web-server to retrieve and display the repeatedly occurring neighbourhood of a gene. *Nucleic Acids Res.* 28 3442–3444. 10.1093/nar/28.18.3442 10982861 PMC110752

[B48] Srebrow A. Kornblihtt A. R. (2006). The connection between splicing and cancer. *J. Cell Sci.* 119 2635–2641. 10.1242/jcs.03053 16787944

[B49] Su F. Yang S. Wang H. Qiao Z. Zhao H. Qu Z. (2020). CIRBP Ameliorates Neuronal amyloid toxicity via antioxidative and antiapoptotic pathways in primary cortical neurons. *Oxid. Med. Cell Longev.* 2020:27 86139. 10.1155/2020/2786139 PMC7063194 32184914

[B50] Sveen A. Kilpinen S. Ruusulehto A. Lothe R. A. Skotheim R. I. (2016). Aberrant RNA splicing in cancer; expression changes and driver mutations of splicing factor genes. *Oncogene* 35 2413–2427. 10.1038/onc.2015.318 26300000

[B51] Tang Z. Li C. Kang B. Gao G. Li C. Zhang Z. (2017). GEPIA: a web server for cancer and normal gene expression profiling and interactive analyses. *Nucleic Acids Res.* 45 W98–W102. 28407145 10.1093/nar/gkx247 PMC5570223

[B52] Uhlen M. Fagerberg L. Hallstrom B. M. Lindskog C. Oksvold P. Mardinoglu A. (2015). Proteomics. Tissue-based map of the human proteome. *Science* 347:1260419. 10.1126/science.1260419 25613900

[B53] Vasaikar S. V. Straub P. Wang J. Zhang B. (2018). LinkedOmics: analyzing multi-omics data within and across 32 cancer types. *Nucleic Acids Res.* 46 D956–D963. 29136207 10.1093/nar/gkx1090 PMC5753188

[B54] Wang C. Zheng M. Wang S. Nie X. Guo Q. Gao L. (2019). Whole genome analysis and prognostic model construction based on alternative splicing events in endometrial cancer. *Biomed. Res. Int.* 2019:2686875. 10.1155/2019/2686875 PMC6634061 31355251

[B55] Wang E. T. Sandberg R. Luo S. Khrebtukova I. Zhang L. Mayr C. (2008). Alternative isoform regulation in human tissue transcriptomes. *Nature* 456 470–476. 10.1038/nature07509 18978772 PMC2593745

[B56] Wu H. Y. Peng Z. G. He R. Q. Luo B. Ma J. Hu X. H. (2019). Prognostic index of aberrant mRNA splicing profiling acts as a predictive indicator for hepatocellular carcinoma based on TCGA SpliceSeq data. *Int. J. Oncol.* 55 425–438. 31268164 10.3892/ijo.2019.4834 PMC6615926

[B57] Xing S. Li Z. Ma W. He X. Shen S. Wei H. (2019). DIS3L2 promotes progression of hepatocellular carcinoma via hnrnp u-mediated alternative splicing. *Cancer Res.* 79 4923–4936. 10.1158/0008-5472.can-19-0376 31331910

[B58] Yamashita Y. Nishiumi S. Kono S. Takao S. Azuma T. Yoshida M. (2017). Differences in elongation of very long chain fatty acids and fatty acid metabolism between triple-negative and hormone receptor-positive breast cancer. *BMC Cancer* 17:589. 10.1186/s12885-017-3554-4 28851309 PMC5576271

[B59] Yen M. C. Chou S. K. Kan J. Y. Kuo P. L. Hou M. F. Hsu Y. L. (2018). Solute Carrier family 27 member 4 (SLC27A4) enhances cell growth, migration, and invasion in breast cancer cells. *Int. J. Mol. Sci.* 19:3434. 10.3390/ijms19113434 30388870 PMC6274775

[B60] Yin Z. Q. Liu J. J. Xu Y. C. Yu J. Ding G. H. Yang F. (2014). A 41-gene signature derived from breast cancer stem cells as a predictor of survival. *J. Exp. Clin. Cancer Res.* 33:49. 10.1186/1756-9966-33-49 24906694 PMC4229870

[B61] Zhang D. Duan Y. Cun J. Yang Q. (2019). Identification of prognostic alternative splicing signature in breast carcinoma. *Front. Genet.* 10:278. 10.3389/fgene.2019.00278 30984247 PMC6448481

[B62] Zhang X. Lan Y. Xu J. Quan F. Zhao E. Deng C. (2019). CellMarker: a manually curated resource of cell markers in human and mouse. *Nucleic Acids Res.* 47 D721–D728. 30289549 10.1093/nar/gky900 PMC6323899

[B63] Zhao D. Zhang C. Jiang M. Wang Y. Liang Y. Wang L. (2020). Survival-associated alternative splicing signatures in non-small cell lung cancer. *Aging* 12 5878–5893. 32282333 10.18632/aging.102983 PMC7185095

[B64] Zhong P. Huang H. (2017). Recent progress in the research of cold-inducible RNA-binding protein. *Future Sci. OA* 3:FSO246. 10.4155/fsoa-2017-0077 PMC5674272 29134130

[B65] Zhu X. Buhrer C. Wellmann S. (2016). Cold-inducible proteins CIRP and RBM3, a unique couple with activities far beyond the cold. *Cell Mol. Life Sci.* 73 3839–3859. 27147467 10.1007/s00018-016-2253-7 PMC5021741

