# Supplementary figures and images for: The Construction of Bone Metastasis-Specific Prognostic Model and Co-expressed Network of Alternative Splicing in Breast Cancer
Source: Front Cell Dev Biol. 2020 Aug 25;8:790. doi: 10.3389/fcell.2020.00790 (PMC7477087; doi:10.3389/fcell.2020.00790)

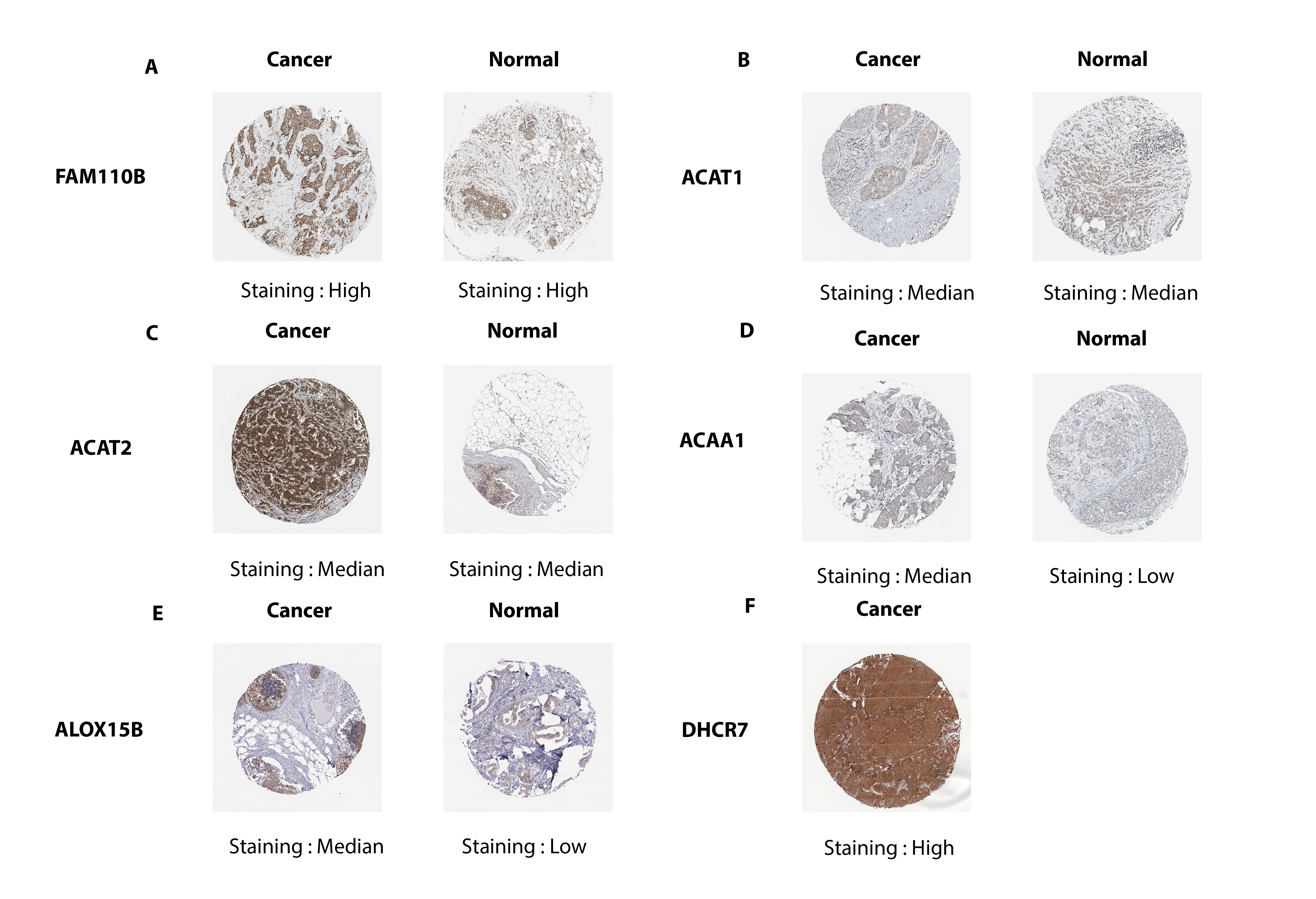

Supplement: FIGURE S1 — External validation of FAM110B (A), ACAT1 (B), ACAT2 (C), ACAA1 (D), ALOX15B (E), and DHCR7 (F) by the Human Protein Atlas. [file Image_1.TIF]

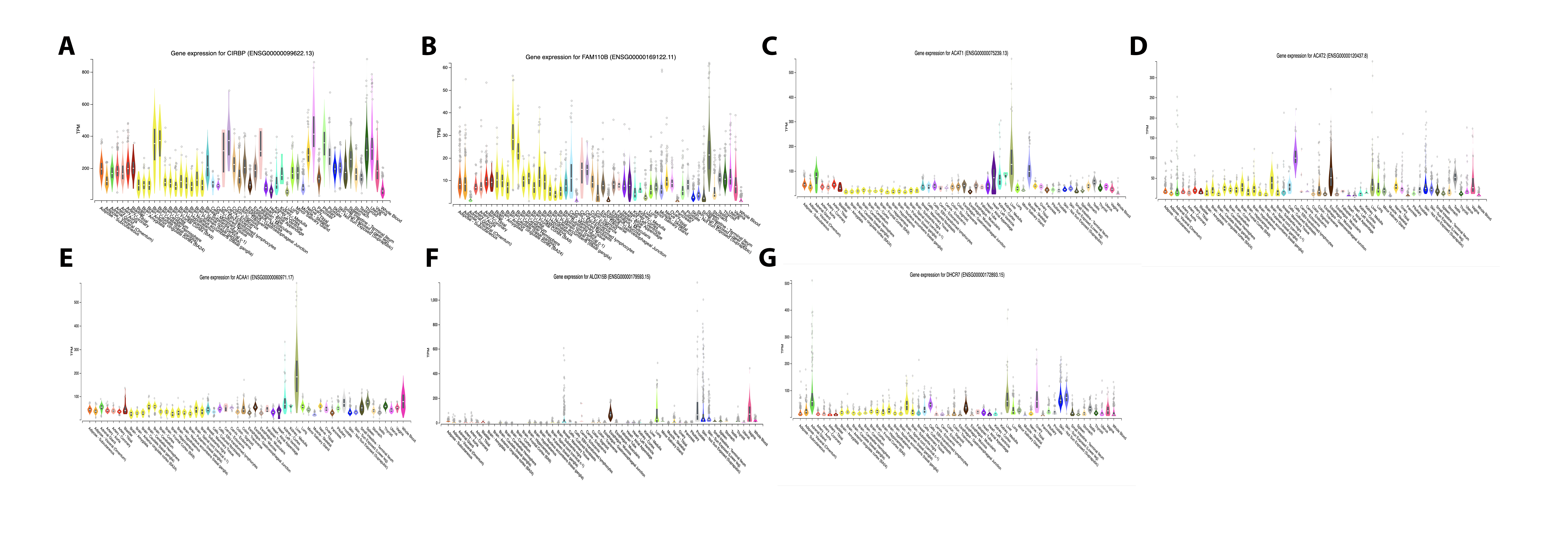

Supplement: FIGURE S2 — External validation of CIRBP (A), FAM110B (B), ACAT1 (C), ACAT2 (D), ACAA1 (E), ALOX15B (F), and DHCR7 (G) by GTEx. [file Image_2.TIF]

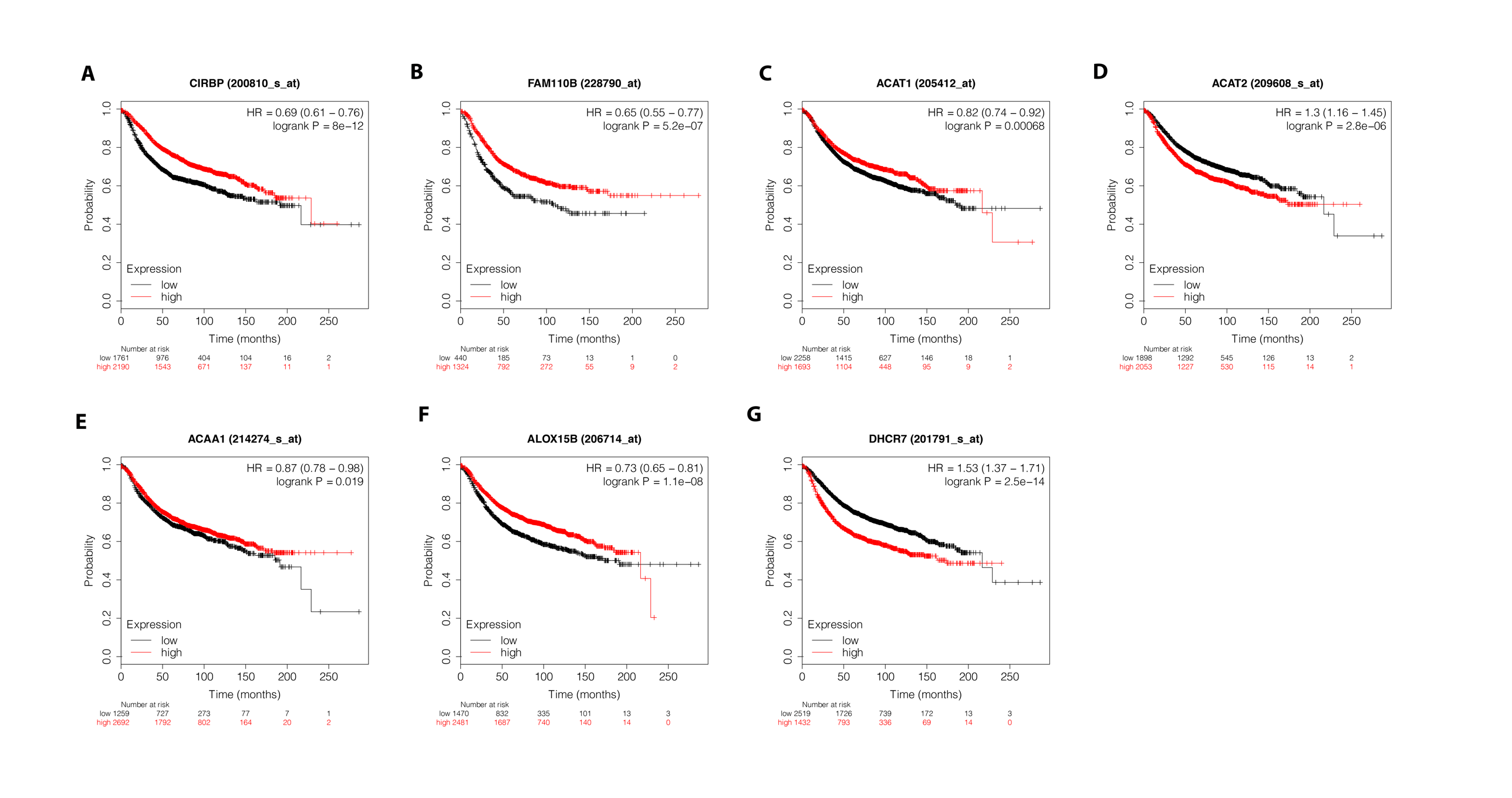

Supplement: FIGURE S5 — External validation of CIRBP (A), FAM110B (B), ACAT1 (C), ACAT2 (D), ACAA1 (E), ALOX15B (F), and DHCR7 (G) by The Kaplan Meier plotter. CIRBP, FAM110B, ACAT1, ALOX15B, and ACAA1 high-expressed in tissue level in BRCA; while ACAT2 and DHCR7 low-expressed in tissue level in BRCA. [file Image_5.TIF]

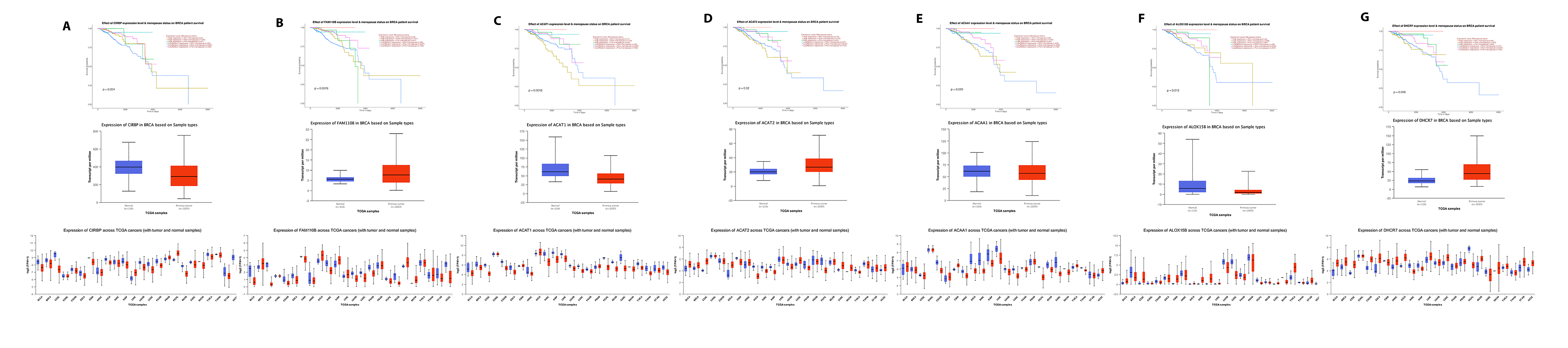

Supplement: FIGURE S6 — External validation of CIRBP (A), FAM110B (B), ACAT1 (C), ACAT2 (D), ACAA1 (E), ALOX15B (F), and DHCR7 (G) by UALCAN. ALOX15B expressed lowly in BRCA; CIRBP, FAM110B, ACAT1, ACAT2, ACAA1, and DHCR7 expressed highly in normal thyroid and BRCA. [file Image_6.TIF]

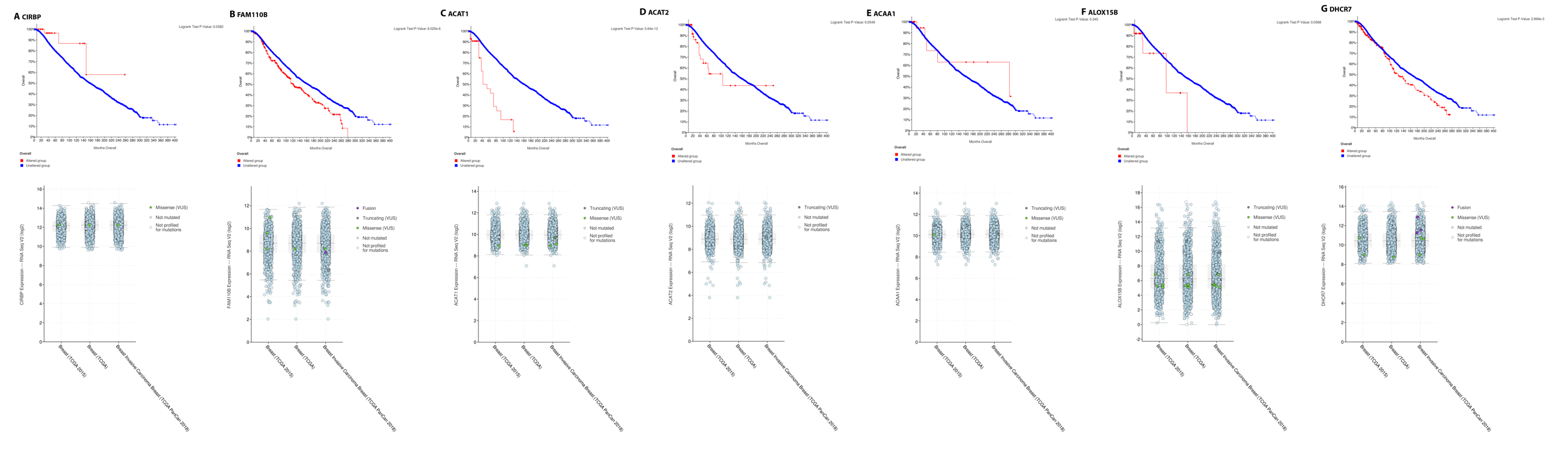

Supplement: FIGURE S8 — External validation of CIRBP (A), FAM110B (B), ACAT1 (C), ACAT2 (D), ACAA1 (E), ALOX15B (F), and DHCR7 (G) by cBioportal. [file Image_8.TIF]

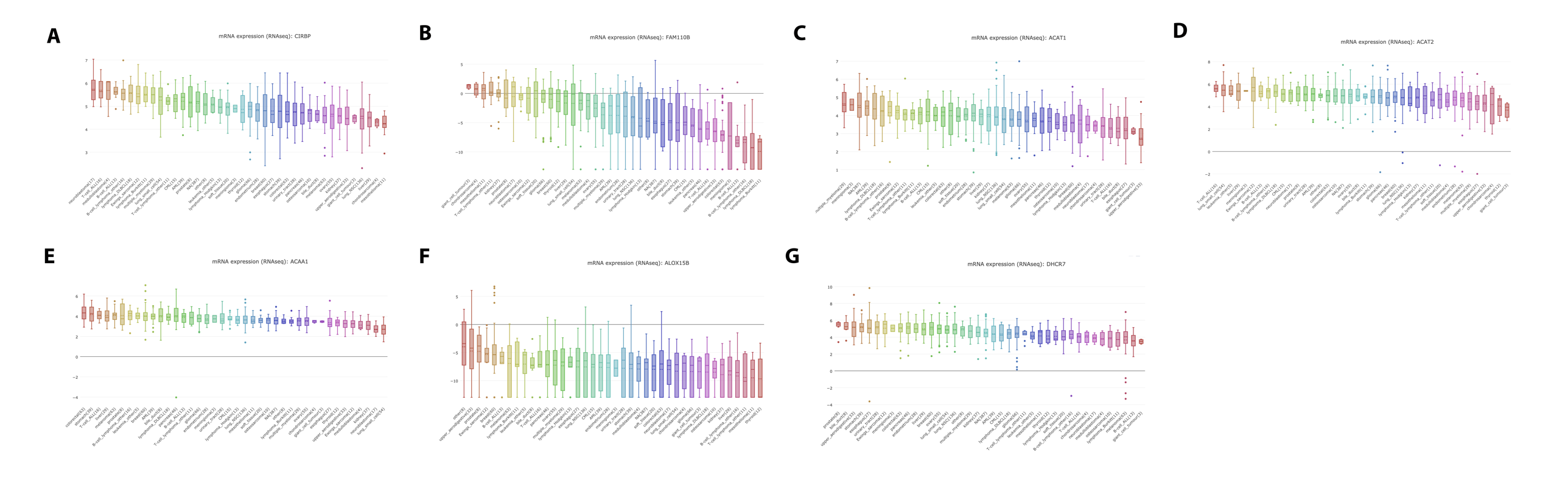

Supplement: FIGURE S10 — External validation of CIRBP (A), FAM110B (B), ACAT1 (C), ACAT2 (D), ACAA1 (E), ALOX15B (F), and DHCR7 (G) by CCLE. FAM110B and ALOX15B low-expressed, while CIRBP, ACAT1, ACAT2, ACAA1, and DHCR7 high-expressed in cellular level in BRCA. [file Image_10.TIF]

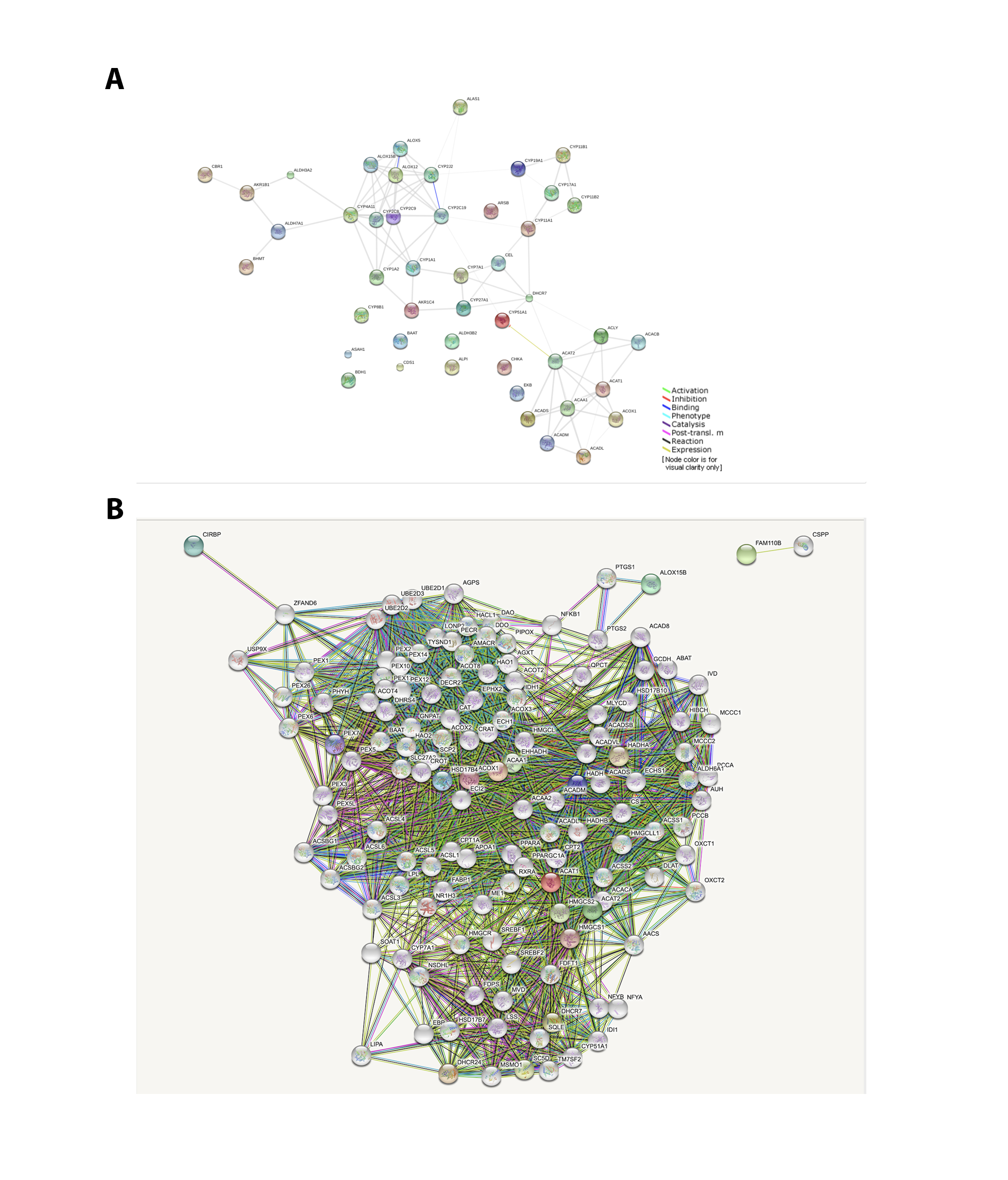

Supplement: FIGURE S11 — The protein-protein interaction networks. (A) The protein network of fatty acid metabolism pathway from Pathcards. (B) The protein network of CIRBP, FAM110B, ACAT1, ACAT2, ACAA1, ALOX15B, and DHCR7 from String. [file Image_11.TIF]
