# Supplementary material for: The Construction of Bone Metastasis-Specific Prognostic Model and Co-expressed Network of Alternative Splicing in Breast Cancer
Source: Front Cell Dev Biol. 2020 Aug 25;8:790. doi: 10.3389/fcell.2020.00790 (PMC7477087; doi:10.3389/fcell.2020.00790)
Supplement: TABLE S1 — Description of patients’ dataset. [file Table_1.DOCX]

**Table S1** Description of Patients’ Dataset.

| **Variables** | **Total Patients (N = 1097)** |
| --- | --- |
| **Age,years** |  |
| Mean SD | 58.46 13.22 |
| Median (Range) | 58 ( 26 - 90 ) |
| **Gender** |  |
| Female | 1085 |
| Male | 12 |
| **stage** |  |
| stage I | 183 |
| stage II | 621 |
| stage III | 249 |
| stage IV | 20 |
| stage X | 13 |
| unknow | 11 |
| **T** |  |
| T1 | 281 |
| T2 | 635 |
| T3 | 138 |
| T4 | 40 |
| TX | 3 |
| **M** |  |
| M0 | 912 |
| M1 | 22 |
| MX | 163 |
| **N** |  |
| N0 | 516 |
| N1 | 364 |
| N2 | 120 |
| N3 | 77 |
| NX | 20 |
| **Bone Metastasis** |  |
| yes | 33 |
| no | 1064 |
| **Distant Metastasis** |  |
| yes | 65 |
| no | 1032 |
