# Supplementary material for: The Construction of Bone Metastasis-Specific Prognostic Model and Co-expressed Network of Alternative Splicing in Breast Cancer
Source: Front Cell Dev Biol. 2020 Aug 25;8:790. doi: 10.3389/fcell.2020.00790 (PMC7477087; doi:10.3389/fcell.2020.00790)
Supplement: TABLE S2 — Number of ASEs and number of ASEs in different ASE type. [file Table_2.DOCX]

**Table S2** Number of ASEs and Number of ASEs in different ASE type

| **ASE type** | **Number of ASEs** | **Number of** |
| --- | --- | --- |
| **Alternate acceptor (AA)** | 444 | 396 |
| **Alternate donor (AD)** | 414 | 374 |
| **Alternate promoter (AP)** | 1,304 | 1,304 |
| **Alternate terminator (AT)** | 251 | 117 |
| **Exon skip (ES)** | 2,728 | 1,801 |
| **Mutually exclusive exons (ME)** | 38 | 37 |
| **Retained intron (RI)** | 254 | 229 |
| **Total** | 5,433 | 2,933 |
