# Supplementary material for: The Construction of Bone Metastasis-Specific Prognostic Model and Co-expressed Network of Alternative Splicing in Breast Cancer
Source: Front Cell Dev Biol. 2020 Aug 25;8:790. doi: 10.3389/fcell.2020.00790 (PMC7477087; doi:10.3389/fcell.2020.00790)
Supplement: TABLE S3 — The external validation of CIRBP, FAM110B, ACAT1, ACAT2, ACAA1, ALOX15B, and DHCR7. [file Table_3.DOCX]

**Table S3** Summary of multidimensional external validation results base on multiple databases

|  | **CIRBP** | | **FAM110B** | | **ACAT1** | | **ACAT2** | | **ACAA1** | | **ALOX15B** | | **DHCR7** | | **Results** |
| --- | --- | --- | --- | --- | --- | --- | --- | --- | --- | --- | --- | --- | --- | --- | --- |
|  | **N** | **B** | **N** | **B** | **N** | **B** | **N** | **B** | **N** | **B** | **N** | **B** | **N** | **B** |  |
| **The human protein atlas** | **NA** | **NA** | **↑** | **↑** | **-** | **-** | **-** | **-** | **↓** | **-** | **↓** | **-** | **NA** | **↑** | FAM110B expressed highly in normal thyroid and BRCA; DHCR7 high-expressed in BRCA, while ACAA1 and ALOX15B low-expressed in normal thyroid (Figure S1). |
| **GTEx** | **-** | **NA** | **-** | **NA** | **-** | **NA** | **↓** | **NA** | **-** | **NA** | **-** | **NA** | **-** | **NA** | ACAT2 low-expressed in normal thyroid (Figure S2). |
| **PROGgeneV2** | **NA** | **↑** | **NA** | **↑** | **NA** | **↑** | **NA** | **↓** | **NA** | **↑** | **NA** | **-** | **NA** | **↓** | CIRBP, FAM110B, ACAT1 and ACAA1 high-expressed in tissue level in BRCA; while ACAT2 and DHCR7 low-expressed in tissue level in BRCA (Figure S3). |
| **GEPIA** | **↑** | **↑** | **-** | **↑** | **↑** | **↑** | **↑** | **-** | **↑** | **↑** | **-** | **↓** | **↑** | **↑** | FAM110B expressed highly in BRCA; ALOX15B expressed lowly in BRCA; ACAT2 expressed highly in normal thyroid; CIRBP, ACAT1, ACAA1 and DHCR7 expressed highly in normal thyroid and BRCA (Figure S4). |
| **The Kaplan Meier plotter** | **NA** | **↑** | **NA** | **↑** | **NA** | **↑** | **NA** | **↓** | **NA** | **↑** | **NA** | **↑** | **NA** | **↓** | CIRBP, FAM110B, ACAT1, ALOX15B and ACAA1 high-expressed in tissue level in BRCA; while ACAT2 and DHCR7 low-expressed in tissue level in BRCA (Figure S5). |
| **UALCAN** | **↑** | **↑** | **↑** | **↑** | **↑** | **↑** | **↑** | **↑** | **↑** | **↑** | **-** | **↓** | **↑** | **↑** | ALOX15B expressed lowly in BRCA; CIRBP, FAM110B, ACAT1, ACAT2, ACAA1 and DHCR7 expressed highly in normal thyroid and BRCA (Figure S6). |
| **Linkedomics** | **NA** | **-** | **NA** | **↓** | **NA** | **-** | **NA** | **-** | **NA** | **-** | **NA** | **-** | **NA** | **-** | FAM110B low-expressed in tissue level in BRCA (Figure S7). |
| **cBioportal** | **NA** | **↑** | **NA** | **↑** | **NA** | **↑** | **NA** | **↑** | **NA** | **↑** | **NA** | **↑** | **NA** | **↑** | CIRBP, FAM110B, ACAT1, ACAT2, ACAA1, ALOX15B and DHCR7 high-expressed in tissue level in BRCA (Figure S8). |
| **Oncomine** | **NA** | **-** | **NA** | **↑** | **NA** | **↓** | **NA** | **↓** | **NA** | **-** | **NA** | **↑** | **NA** | **↑** | ACAT1 and ACAT2 low-expressed, while FAM110B, ALOX15B and DHCR7 high-expressed in tissue level in BRCA (Figure S9). |
| **CCLE** | **NA** | **↑** | **NA** | **↓** | **NA** | **↑** | **NA** | **↑** | **NA** | **↑** | **NA** | **↓** | **NA** | **↑** | FAM110B and ALOX15B low-expressed, while CIRBP, ACAT1, ACAT2, ACAA1 and DHCR7 high-expressed in tissue level in BRCA (Figure S10). |

Note: “N” was defined as normal; “B” was defined as Breast cancer;“↑” was defined as a significantly high-expressed gene; “↓” was defined as a significantly low-expressed gene; “NA” was defined as “Not available”; “-” was defined as a gene with no significant difference in expression.

Abbreviations: BRCA, breast cancer; GTEx, Genotype-Tissue Expression; CCLE, Cancer Cell Line Encyclopedia; GEPIA, Gene Expression Profilling Interactive Analysis.
