# Supplementary material for: The Construction of Bone Metastasis-Specific Prognostic Model and Co-expressed Network of Alternative Splicing in Breast Cancer
Source: Front Cell Dev Biol. 2020 Aug 25;8:790. doi: 10.3389/fcell.2020.00790 (PMC7477087; doi:10.3389/fcell.2020.00790)
Supplement: TABLE S4 — Summary of external validation. [file Table_4.DOCX]

**Table S4** Summary of External Validation

| **Database** | **CIRBP** | **FAM110B** | **ACAT1** | **ACAT2** | **ACAA1** | **ALOX15B** | **DHCR7** |
| --- | --- | --- | --- | --- | --- | --- | --- |
|  | **(splicing factor gene)** | **(alternative splicing events gene)** | **(pathway gene)** | **(pathway gene)** | **(pathway gene)** | **(pathway gene)** | **(pathway gene)** |
| **The human protein atlas** | Normal NA | Normal high | Normal median | Normal median | Normal low | Normal low | Normal NA |
|  | Cancer NA | Cancer high | Cancer median | Cancer median | Cancer median | Cancer median | Cancer high |
| **GTEx** | Normal median | Normal median | Normal median | Normal low | Normal median | Normal median | Normal median |
| **PROGgeneV2** | K-M P<0.001 | K-M P=0.043 | K-M P=0.003 | K-M P<0.001 | K-M P<0.001 | K-M P=0.010 | K-M P<0.001 |
| **GEPIA** | Normal high | Normal median | Normal high | Normal high | Norma high | Normal median | Normal high |
|  | Tumor high | Tumor high | Tumor high | Tumor median | Tumor high | Tumor low | Tumor high |
|  | K-M: P=0.01 | K-M: P=0.42 | K-M: P=0.12 | K-M: P=0.69 | K-M: P=0.86 | K-M: P=0.15 | K-M: P=0.47 |
|  | Stage: p=0.040 | Stage: p=0.119 | Stage: p=0.255 | Stage: p=0.476 | Stage: p=0.028 | Stage: p=0.402 | Stage: p=0.245 |
| **The Kaplan Meier plotter** | K-M: P<0.001 | K-M: P<0.001 | K-M: P<0.001 | K-M: P<0.001 | K-M: P=0.019 | K-M: P<0.001 | K-M: P<0.001 |
| **UALCAN** | Normal high | Tumor high | Normal high | Tumor high | Tumor high | Tumor median | Tumor high |
|  | Tumor high | Normal high | Tumor high | Normal high | Normal high | Normal low | Normal high |
|  | K-M: P=0.004 | K-M: P=0.008 | K-M: P=0.002 | K-M: P=0.020 | K-M: P=0.029 | K-M: P=0.013 | K-M: P=0.026 |
| **Linkedomics** | K-M: P=0.0645 | K-M: P<0.001 | K-M: P<0.001 | K-M: P=0.193 | K-M: P=0.932 | K-M: P=0.016 | K-M: P=0.842 |
| **cBioportal** | K-M: P=0.045 | K-M: P<0.001 | K-M: P<0.001 | K-M: P=0.055 | K-M: P=0.345 | K-M: P=0.057 | K-M: P=0.003 |
|  | Tumor high | Tumor high | Tumor high | Tumor high | Tumor high | Tumor high | Tumor high |
| **Oncomine** | Over-expression: | Over-expression: | Over-expression: | Over-expression: | Over-expression: | Over-expression: | Over-expression: |
|  | P = 0.244 | P = 0.005 | P = 0.898 | P = 0.649 | P = 0.331 | P = 0.151 | P = 0.327 |
|  | Under-expression: | Under-expression: | Under-expression: | Under-expression: | Under-expression: | Under-expression: | Under-expression: |
|  | P = 0.272 | P = 0.638 | P = 0.183 | P = 0.138 | P = 0.584 | P = 0.566 | P = 0.803 |
| **CCLE** | Tumor high | Tumor low | Tumor high | Tumor high | Tumor high | Tumor low | Tumor high |
